# Antifungal Activity of *Streptomyces* sp. F204 Against Rhizoctonia Solani and Whole-Genome Sequencing and Metabolomic Analysis

**DOI:** 10.3390/jof12100782

**Published:** 2026-10-19

**Authors:** Longhui Shen, Jinyan Xue, Linyan Zhu

**Affiliations:** 1 Department of Pharmacy, The Affiliated Women and Children’s Hospital of Ningbo University, Ningbo 315000, China; nbslh22@163.com; 2 School of Pharmacy, Liaoning University of Traditional Chinese Medicine, 77 Shenming Road, Dalian 116600, China; xuejy127td@163.com

**Keywords:** *Streptomyces* F204, *Rhizoctonia solani* Kühn, active compound, genome, metabolite

## Abstract

*Rhizoctonia solani* Kühn is a destructive phytopathogenic fungus with an exceptionally broad host range, infecting over 32 plant families and 188 genera. In this study, we evaluated the antifungal activity of compound **1** from *Streptomyces* sp. F204 against *R. solani*, and characterized its genomic features and metabolomic responses under different culture conditions. Based on whole-genome sequences, the highest average nucleotide identity (ANI) and digital DNA–DNA hybridization (dDDH) values between strain F204 and its closest related type strain, *Streptomyces puniciscabiei* S77, were 91.64% and 45.00%, respectively. Compound **1**, the active metabolite isolated from the fermentation broth of *Streptomyces* sp. F204, was evaluated using the mycelial growth rate method. Genome annotation predicted 8022 genes in strain F204, including protein-coding genes, rRNA genes, and tRNA genes, covering 86.72% of the total genome length. Additionally, antiSMASH 6.0.0 analysis identified 23 biosynthetic gene clusters putatively involved in secondary metabolite production. Comparative metabolomics revealed significantly enriched pathways across different media, including nicotinate and nicotinamide metabolism in Medium No. 2 (B) versus Gause’s synthetic medium (A), and pyrimidine metabolism as well as alanine, aspartate, and glutamate metabolism in ISP4 medium (C) versus Gause’s synthetic medium (A). On the basis of NMR and mass spectrometry (MS) data, compound **1** is proposed to be a structural analog of daptomycin. It exhibited strong concentration-dependent inhibition of *R. solani* mycelial growth, achieving 93.07 ± 0.49% inhibition at 200 μg/mL, with an IC_50_ value of 14.9 μg/mL. These findings indicate that *Streptomyces* sp. F204 represents a promising source of antifungal metabolites with considerable biosynthetic potential.

## 1. Introduction

*Rhizoctonia solani* Kühn is a destructive phytopathogenic fungus with an exceptionally broad host range, infecting over 32 families and 188 genera of plants, including cereals, legumes, vegetables, and various other economically important crops [1,2]. Its pathogenic mechanisms likely differ across plant species. The fungus infects rice at all growth stages, causing seed rot, seedling sheath blight, and eventual plant death [3]. In addition, it induces stem rot in tomatoes; hypocotyl and root rot in spinach, broccoli, and melons; and bottom rot in lettuce [4]. Chemical control has long been the primary strategy for managing this disease. However, the prolonged and excessive use of chemical pesticides not only results in pesticide residues and environmental pollution but also accelerates the development of fungicide resistance in the pathogen population, leading to progressively declining field efficacy. Consequently, the development of environmentally friendly bio-based fungicides with novel modes of action has become an important research direction.

Microbial secondary metabolites represent a prolific source of biologically active natural products. Over the long course of evolution, microorganisms have developed highly complex secondary metabolic systems capable of producing structurally diverse and functionally rich active molecules. It is estimated that antibiotics produced by *Streptomyces* account for approximately 70–80% of all naturally occurring bioactive products [5,6]. The genus *Streptomyces* is one of the most important producers of natural cyclic peptide antibiotics. The cyclic peptide compounds synthesized by *Streptomyces* are chemically diverse, encompassing cyclic depsipeptides, cyclic lipopeptides, and other types. Antimycins are a class of cyclic depsipeptides with a nine-membered bislactone ring skeleton produced by *Streptomyces*. They specifically bind to the cytochrome bc_1_ complex in the inner mitochondrial membrane, inhibiting electron transfer in the mitochondrial respiratory chain, thereby exerting antifungal, insecticidal, and antitumor activities. To date, more than twenty natural antimycin subtypes have been discovered [7,8,9]. Daptomycin, a cyclic lipopeptide antibiotic produced by *Streptomyces roseosporus*, disrupts cell membrane function through a calcium-dependent membrane insertion mechanism, rapidly killing a broad range of Gram-positive bacteria [10].

In recent years, increasing attention has been directed toward the application of *Streptomyces* and their metabolites for biological control of rice sheath blight. For instance, *Streptomyces olivoreticuli* ZZ-21, isolated from forest inter-root soil, exhibits a significant inhibitory effect against *R. solani*, and its active antifungal components are preliminarily identified as peptide-type compounds [11]. Another *Streptomyces* sp. PM5 produce antifungal compounds SPM5C-1 and SPM5C-2, which reduce sheath blight development by 82.3% in greenhouse trials on rice [12]. These findings collectively highlight the potential of *Streptomyces* strains as sources of cyclic lipopeptide-type antifungal agents against *R. solani*.

*Streptomyces* genomes harbor immense potential for the biosynthesis of diverse bioactive secondary metabolites. With the advent of genome sequencing technologies, it has become evident that these genomes typically contain dozens, or even more, biosynthetic gene clusters (BGCs), which theoretically endow the strains with a metabolic capacity far exceeding that observed under standard laboratory conditions. However, secondary metabolite production in *Streptomyces* is not constitutive; rather, it is profoundly influenced by cultivation parameters. Under conventional conditions, most BGCs remain transcriptionally silent, and their cognate metabolites are rarely detected. By strategically modifying the culture environment, however, these silent clusters can be activated, prompting the same strain to produce a distinct array of secondary metabolites. This principle is encapsulated in the “ One Strain, Many Compounds” (OSMAC) strategy, which centers on systematically varying fermentation parameters, including medium composition, carbon and nitrogen sources, supplementation with amino acid precursors or metal ions, and adjustments to temperature and pH to expose the strain to diverse growth conditions, thereby triggering the expression of different BGCs [13]. The profound impact of culture conditions on the metabolomic output of *Streptomyces* has been corroborated by numerous studies. For instance, *Streptomyces cavourensis* NA4 produce only bafilomycins in a standard medium; however, when glycerol is replaced with wheat bran, the biosynthetic gene cluster for PTM-type frontalamides is successfully activated, leading to the identification of seven major PTM compounds via LC-MS analysis [14]. Collectively, these findings underscore the effectiveness of culture condition modulation as a strategy for discovering novel antibiotics.

In this study, a *Streptomyces* strain isolated from the rhizosphere soil of an ancient banyan tree was designated as F204 based on 16S rDNA sequence and whole-genome analysis. Experiments showed that the fermentation filtrate of strain F204 exhibited pronounced antagonistic activity against *R. solani.* We further undertook the isolation of the bioactive components, investigated the effects of different culture conditions on metabolite production, and performed whole-genome analysis, aiming to provide a scientific basis for the further development and utilization of this strain.

## 2. Materials and Methods

### 2.1. Materials

Gause’s synthetic medium (Gause’s No. 1 medium) and potato dextrose agar (PDA) were purchased from Beijing Aoboxing Biotechnology Co., Ltd. (Beijing, China). Silica gel GF254 plates and silica gel (200–300 mesh) were procured from Qingdao Marine Chemical Ltd. (Qingdao, China). Sephadex LH-20 (Amersham Biosciences, Uppsala, Sweden) was utilized for column chromatography.

- Gause’s synthetic medium: 20 g soluble starch, 0.5 g NaCl, 1 g KNO_3_, 0.5 g K_2_HPO_4,_ 0.5 g MgSO_4_•7H_2_O, 0.01 g FeSO_4_•7H_2_O (per liter of distilled water). Adjust the pH to 7.0–7.2.
- Medium No. 2: 10 g corn meal, 10 g glucose, 10 g peptone, 5 g yeast extract, 2 g calcium carbonate (per liter of distilled water). Adjust the pH to 7.0–7.4.
- ISP4 medium: 10 g soluble starch, 1 g K_2_HPO_4_ (anhydrous), 1 g MgSO_4 •_7H_2_O, 1 g NaCl, 2 g (NH_4_)_2_SO_4_, 2 g CaCO_3_ (per liter of distilled water).

A trace salts stock solution was prepared by dissolving 0.1 g each of FeSO_4_•7H_2_O, MnCl_2_•4H_2_O, and ZnSO_4_•7H_2_O in 100 mL of distilled water.

The pH was adjusted so that it was between 7.0 and 7.4.

### 2.2. Culture Conditions

*Streptomyces* sp. F204, a bacterium isolated from the rhizosphere soil of an ancient banyan tree planted approximately 500 years ago in Jian city, Jiangxi province, China, was deposited in the Microbiology and Immunology Laboratory at Ningbo University (Ningbo, China). This strain exhibited broad-spectrum antibacterial activity when first isolated in May 2019. The bacterial strain was maintained in 50% glycerol at −80 °C and routinely cultured on Gause’s Synthetic Agar medium plates.

*Rhizoctonia solani* Kühn used in this study was also obtained from the Microbiology and Immunology Laboratory, Ningbo University. The fungus was stored in sterile tubes containing PDA and routinely grown on PDA plates.

### 2.3. Identification of the Bacterium

The strain was identified based on morphological, physiological, biochemical, and cultural characteristics, according to the taxonomic criteria of Bergey’s Manual of Determinative Bacteriology [15]. Cellular morphology was observed under a microscope. For molecular identification, the 16S rRNA gene was amplified by PCR using universal prokaryotic primers: forward primer 5′-AGAGTTTGATCMTGG CTCAG-3′ and reverse primer 5′-GGTTACCTTGTTA CGACTT-3′. Genomic DNA was extracted using a UNIQ-10 column genomic DNA kit (Sangon Biotech, Shanghai, China). The PCR product was sequenced by a commercial automated sequencing system (Sangong Biotech Ltd., Shanghai, China). The 16S rRNA gene sequencing of F204 was performed according to the previous method [16]. The obtained sequence was compared with the available 16S rRNA gene sequences of validly named species at the EzBioCloud server (http://www.ezbiocloud.net/, accessed on 30 July 2026). A phylogenetic tree was constructed with the neighbor-joining algorithm using the software package MEGA 11.0. Bootstrap analysis was performed with 1000 replications.

### 2.4. Rhizoctonia Solani Activation

Fresh fungal mycelial plugs (10 mm) were taken from the edge of the colony, inoculated onto PDA solid medium, and cultured at 28 °C for 3 d. The activated fungal cultures were obtained after two successive transfers.

### 2.5. Purification and Structural Identification of Compound from Streptomyces sp. F204

*Streptomyces* sp. F204 was inoculated into a 250 mL conical flask containing Gause’s synthetic medium to prepare a seed suspension. The seed suspension was then inoculated at 2% (*v*/*v*) into 500 mL conical flasks each containing 200 mL of Gause’s synthetic fermentation medium, with a total volume of 50 L. The cultures were incubated at 30 °C with shaking at 180 rpm for 5 d. After incubation, the culture broth was centrifuged at 5000× *g* at 25 °C for 10 min and the supernatant was concentrated to 1 L using a vacuum rotary evaporator (EYELA, Tokyo, Japan) at 50 °C and 50 rpm. An equal volume of ethyl acetate was added to the concentrate, and the mixture was extracted three times. The combined organic phases were evaporated at 45 °C to obtain the crude extract. For column chromatography (CC), the crude extract was mixed with silica gel 1:1, w/w, stirred, and dissolved with methanol. The mixture was dried and ground into a fine powder before being loaded onto a silica gel column (200–300 mesh). The extract was subjected to silica gel column chromatography. The column (5 cm i.d. ×80 cm) was packed with silica gel (200–300 mesh) at a silica gel-to-sample ratio of approximately 50:1 (*w*/*w*). Stepwise elution was performed with a gradient of chloroform and methanol (100:1 to 50:50, v/v). Fractions of approximately 50 mL were collected and monitored by thin-layer chromatography (TLC) on silica gel plates. Fractions showing identical TLC profiles were combined to yield major fractions. The fractions were further assayed for anti-*Rhizoctonia solani* activity. Fractions showing activity were further subjected to purification on a Sephadex LH-20 gel filtration column, which was eluted with a chloroform–methanol mixture 1:1, v/v. Semi-preparative HPLC was employed for the purification of the compounds.

The purified compounds were characterized by positive-ion mode mass spectrometry (MS) and nuclear magnetic resonance (NMR) spectroscopy. MS was performed on an UPLC-QTOf-MS Xevo G3 QTOF (Waters, Milford, MA, USA), and NMR spectra were recorded on a Bruker AV-600 (Bruker, Fällanden, Switzerland) in methanol-d_4_ containing TMS as the internal standard.

### 2.6. Antifungal Activity of Compound **1** Against Rhizoctonia solani

The inhibitory effect of different concentrations of the compound on the mycelial growth of *R. solani* was determined using the mycelial growth rate method [17]. The detailed procedure was as follows. The compound was first dissolved in an appropriate amount of distilled water to prepare a stock solution, which was then diluted with sterilized distilled water under sterile conditions to obtain a 1 mg/mL compound solution. This solution was mixed with PDA medium to achieve final compound concentrations of 200, 100, 50, 25, and 12.5 μg/mL. The mixtures were poured into sterile Petri dishes on a clean bench. A 10 mm mycelial plug of *R. solani* was taken from the edge of an actively growing colony and placed upside down (mycelial side down) at the center of each plate. The plates were sealed with parafilm and incubated at 25 °C. The blank control group was prepared using PDA medium containing an equal amount of sterile water. Following the method of Ishikawa et al. [18], we used validamycin at 100 µg/mL in the subsequent experiments of this study as a positive control. Each treatment was performed in triplicate.

After incubation, the colony diameter of each plate was measured using the criss-cross method. The inhibition rate of mycelial growth was calculated using the following Formula (1):

(1) Inhibition rate (%) = [(C-d)-(S-d)/(C-d)] × 100%

where C is the average colony diameter in the control group, S is the average colony diameter in the compound treatment group, and d is the diameter of the original mycelial plug (10 mm). The average inhibition rate was calculated for each concentration.

The *IC*_50_ value was estimated by log-dose linear interpolation using the two concentrations that bracketed 50% inhibition. The following equation was used:

(2) $$\log_{10}(IC_{50})=\log_{10}(C_{1})+\frac{50-I_{1}}{I_{2}-I_{1}}[\log_{10}(C_{2})-\log_{10}(C_{1})]$$

where (*C*_1_) and (*C*_2_) are the lower and higher concentrations surrounding 50% inhibition, and (*I*_1_) and (*I*_2_) are their corresponding mean inhibition rates.

### 2.7. Whole-Genome Sequencing and Bioinformatics Analysis of Streptomyces sp. F204

#### 2.7.1. Genome Sequencing and Assembly

*Streptomyces* sp. F204, cryopreserved in glycerol in the laboratory, was thawed and inoculated onto Gause’s No. 1 agar medium by the streak plate method. The strain was obtained by repeated streaking on Gause’s No. 1 agar medium and confirmed by colony morphology. A single-colony culture was then incubated at 30 °C in a constant-temperature incubator for 5 d. A well-grown colony of *Streptomyces* sp. F204 was selected, and a piece of the colony was inoculated into a 250 mL conical flask containing 100 mL of Gause’s No. 1 liquid medium. The flask was incubated at 30 °C with shaking at 180 rpm for 5 d. The cultured *Streptomyces* sp. F204 broth was then transferred into 100 mL centrifuge tubes and centrifuged at 5000× *g* for 5 min using a high-speed centrifuge. The supernatant was discarded, leaving only the cell pellet. Under sterile conditions, the cell pellet was transferred into 1 mL centrifuge tubes using forceps. The tubes were wrapped with gauze, treated with liquid nitrogen for cryopreservation, placed in a freezer box, and sent to Wuhan Benagen Tech Solutions Company Limited for whole-genome sequencing of *Streptomyces* sp. F204.

Whole-genome sequencing of *Streptomyces* sp. F204 was performed using both the Nanopore third-generation sequencing platform and second-generation sequencing technology [19]. Genome assembly was performed using a hybrid Nanopore-Illumina strategy. Nanopore raw fast5 files were base-called with Guppy v5.0.16 and filtered using thresholds of Q ≥ 7 and length ≥ 1600 bp. Illumina paired-end reads were filtered with fastp v0.23.2 by removing read pairs containing > 10% N bases or >50% low-quality bases with Q ≤ 5. The filtered Nanopore reads were assembled de novo using Flye v2.9, and the resulting assembly was polished with Illumina reads using Pilon v1.24. Assembly depth was assessed by mapping Illumina reads with BWA v0.7.17 and Nanopore reads with Minimap2 v2.24, followed by depth calculation using Samtools v1.15.1 with a 2000 bp sliding window.

Based on the whole genome sequence of bacteria, using EzBioCloud, the average nucleotide identity (ANI) between the genome of this strain and that of the type strain was calculated. Digital DNA-DNA hybridization (dDDH) values between strain F204 and its closely related type strains were calculated using the Genome-to-Genome Distance Calculator (GGDC) 3.0 server [20]. The 70% dDDH threshold was used for species delineation.

#### 2.7.2. Genome Structure and Genome Circle Map Visualization

Coding genes in the assembled genome were predicted using Prokka software (https://github.com/tseemann/prokka, accessed on 1 July 2025) [21]. Prokka is a suite of tools for predicting various genomic elements: it employs Prodigal for coding gene prediction, Aragorn for tRNA prediction, RNAmmer for rRNA prediction, and Infernal for miscellaneous RNA (miscRNA) prediction. All predicted genomic elements were then integrated and subjected to preliminary annotation. The R package circlize [22] was used to integrate the predicted genomic information of *Streptomyces* sp. F204, including genome structural annotation.

#### 2.7.3. Protein-Coding Gene Functional Annotation

Functional annotation was performed using DIAMOND v2.0.15 and eggNOG-mapper v2.1.9 against four major databases: Kyoto Encyclopedia of Genes and Genomes (KEGG), COG (Clusters of Orthologous Groups), GO (Gene Ontology) and CAZy (Carbohydrate-Active enZYmes Database), with the following parameters: E-value ≤ 1 × 10^−5^ and default settings.

#### 2.7.4. Analysis of Secondary Metabolite Biosynthetic Gene Clusters

The antiSMASH 6.0.0 program was used to predict secondary metabolite gene clusters in the *Streptomyces* genome [23].

### 2.8. Metabolomics

#### 2.8.1. Metabolome Extraction

Metabolite extraction was performed using a slightly modified method described by Hu [24]. Initially, the cell suspension was adjusted to 1 × 10^8^ CFU/mL with sterile saline. It was then added to 100 mL of Gause’s synthetic medium (A), Medium No. 2 (B), and ISP4 medium (C), and incubated at 30 °C and 180 rpm in a shaking incubator for 5 d. Then the culture broth was centrifuged at 5000× *g* at 4 °C for 10 min. The resulting precipitate, which contained mycelium, was harvested. To remove the medium, the mycelium was then washed three times with 0.01 M sterile PBS. Next, the mycelium was quickly immersed in 1 mL of cold (100%) methanol in a centrifuge tube, rotated, and mixed. To halt metabolite activity, the mycelium was flash-frozen in liquid nitrogen. Following this, the mycelium stored in liquid nitrogen was removed and subjected to cell lysis, and the metabolites were then centrifuged at 10,000× *g* for 10 min at 4 °C to collect intracellular metabolites. Each group underwent three biological replicates.

#### 2.8.2. Liquid Chromatography–Mass Spectrometry Analysis

At 4 °C and 10,000× *g*, the sample underwent centrifugation for 3 min. Subsequently, to analyze the sample, the supernatant (200 µL) was transferred to an LC-MS vial. The supernatant was then subjected to a C_18_ column (2.1 mm × 100 mm, 1.8 µm, Waters ACQUITY Premier HSS T3, Waters Corporation, Milford, MA, USA), which was eluted with a linear gradient of mobile phase A that contained ultrapure water, including 0.1% formic acid, and mobile phase B containing acetonitrile, including 0.1% formic acid. This was performed at a flow rate of 0.40 mL/min (Table S7). Chromatographic separation was performed on a Vanquish UHPLC system at 40 °C with an injection volume of 4 μL. Compounds were detected using a Q Exactive HF-X mass spectrometer operating in both positive and negative ion modes. The electrospray ionization voltage was 3500 V for positive-ion mode and 3200 V for negative-ion mode.

#### 2.8.3. Data Processing

Compounds that had an accurate *m*/*z* were annotated. To clarify metabolic pathways, it was utilized for METLIN, KEGG, HMDB, and MetaboAnalyst (version 5.0). After logarithmic transformation, the data were centered (CTR) in SIMCA 16.0.2 (Sartorius Stedim Data Analysis). To detect differentially expressed metabolites, a Student’s *t*-test was applied with a significance threshold set at a *p*-value < 0.05, where there was a parameter such as fold-change (FC) > 2.0 or <0.5. Pathway enrichment analysis was performed using the KEGG database and the hypergeometric test, with a significance threshold of *p* < 0.05.

## 3. Results

### 3.1. Identification of the Bacterium

**Figure 1:**
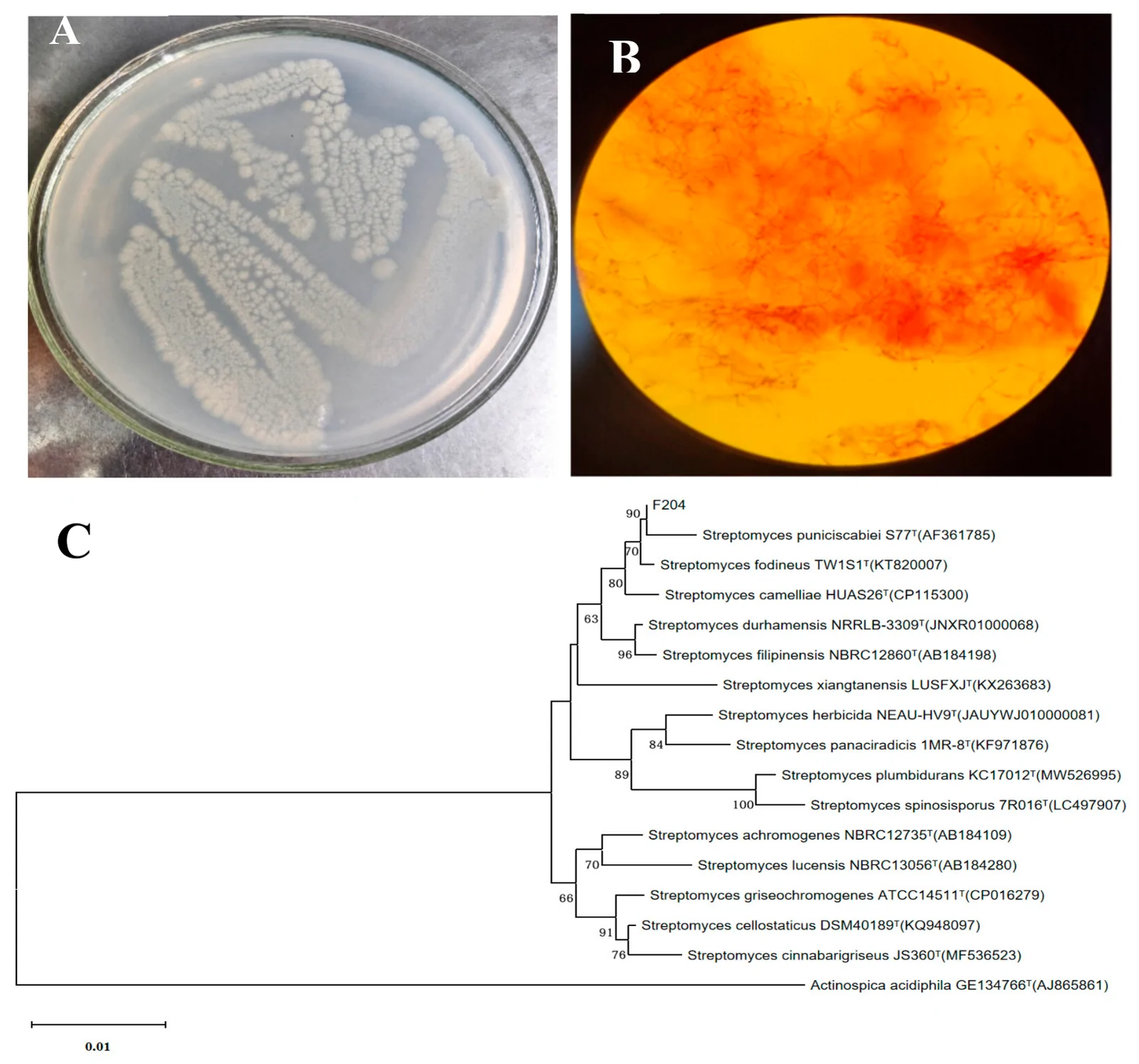
Bacterial strain identification. (**A**) The morphology of the clone. (**B**) Microscopic image. (**C**) Neighbor-joining phylogenetic tree showing the relationship between strain F204 and related taxa based on 16S rRNA gene sequences. Bootstrap values greater than 50% from 1000 replicates are shown at the branch nodes. *Actinospica acidiphila* GE134766 was used as the outgroup. The scale bar represents 0.01 nucleotide substitutions per site.

*Streptomyces* sp. F204 was cultured on Gause‘s No. 1 agar medium at 30 °C for 4–6 d. On the culture plates, the colonies consistently appeared white, dry, slightly convex, and round with a granular surface. Over time, the round colonies gradually enlarged and coalesced. A slide of *Streptomyces* sp. F204 was prepared, stained with crystal violet, and observed under a microscope, revealing that the strain exhibited filamentous hyphal morphology (Figure 1A,B).

#### 3.1.1. Physiological and Biochemical Characteristics of *Streptomyces* sp. F204

Physiological and biochemical tests were conducted on *Streptomyces* sp. F204. The results showed that *Streptomyces* sp. F204 could utilize glucose, sucrose, and D-galactose, but not lactose. It utilized citrate and gave a yellow color in the methyl red test, indicating that the strain produces acid and can survive under slightly acidic conditions. The strain does not utilize urea. In addition, it did not produce H_2_S but can hydrolyze gelatin.

#### 3.1.2. Molecular Identification of *Streptomyces* sp. F204

Sequencing results showed that the 16S rRNA gene sequence of *Streptomyces* sp. F204 was 1472 bp in length. The sequence was deposited in GenBank under accession number OR965865. BLAST (EzBioCloud 16S database version 2025.04.21) analysis of the 16S rRNA gene sequence of strain F204 using the EzBioCloud server showed the highest similarity to *Streptomyces puniciscabiei* S77 (99.65%). Consistently, the 16S rRNA gene-based phylogenetic tree placed strain F204 closest to *S. puniciscabiei* S77. Based on these results, strain F204 was assigned to the genus *Streptomyces* (Figure 1C).

### 3.2. Isolation, Purification, and Structural Elucidation of Compound from Streptomyces sp. F204

The crude products extracted from the broth of *Streptomyces* sp. F204 were further isolated using chromatography. Stepwise elution was performed with a gradient of chloroform and methanol (100:1 to 50:50, v/v) and eleven parts were obtained. Compound was obtained, and its structure was characterized based on spectral data, including NMR and MS.

Compound **1** was obtained from Fraction F11 through bioassay-guided fractionation. Fraction F11, which showed the strongest anti-*Rhizoctonia solani* activity, was therefore further separated by reversed-phase chromatography and Sephadex LH-20 gel filtration column prior to isolation. Semi-preparative HPLC was employed for the purification of compounds.

Compound **1** was isolated as yellow solid (ethanol) (weight, 12.0 mg): UV at λmax (220 nm) was 4.65, 260 nm was 3.98. The structure was characterized by ESI-MS (Figure S1), ^1^H-NMR (CD_3_OD, 600 MHz) (Figure S2) and ^13^C-NMR (CD_3_OD, 150 MHz) (Figure S3). The ESI-MS spectrum displayed a prominent ion peak at m/z 810.8956, corresponding to the most abundant precursor ion, which was assigned to the doubly protonated species [M + 2H]^2^. This observation was attributable to the presence of multiple basic ionizable groups in the daptomycin molecule. The neutral monoisotopic mass derived from this ion (calculated as 810.8956 × 2 − 2 × 1.0073 = 1619.7762 Da) was in good agreement with the theoretical monoisotopic mass of 1619.71037 Da for the molecular formula C_72_H_101_N_17_O_26_.

The structural assignment was further corroborated by the isotopic distribution and MS/MS fragmentation patterns. Characteristic fragment ions were observed, including *m*/*z* 341.2355, which corresponds to the modified residue at position 1 (Trp) bearing a C_9_H_19_CO fatty acyl chain; the calculated mass of this modified residue (C_9_H_19_CO + Trp) is 341.2355. Another diagnostic fragment at m/z 1280.5538 represents the remaining moiety of daptomycin after removal of the C_9_H_19_CO–Trp^1^ residue, with a residue mass of 1280.5538. Additionally, the fragment at m/z 1051.4775 corresponded to the daptomycin core after elimination of the C_9_H_19_CO–Trp^1^–Asn^2^–Asn^3^ segment. Collectively, these fragment ions unambiguously confirmed the daptomycin structure.

In the low-field region, the ^13^C NMR spectrum exhibited one carbonyl carbon signal at δ_C_ 199.9 (attributed to the n-decanoyl chain), seventeen carbonyl carbon signals in the range of δ_C_ 171.1–177.1 (from amino acid residues), and fourteen aromatic carbon signals between δ_C_110.7 and 152.6, corresponding to both methine and quaternary carbons. The DEPT spectrum showed four methyl groups at δ_C_ 14.5, 15.8 and 17.2 and 32.6 and several methylene group carbons in the range of δ_C_ 23.7–44.1 and 63.5 in the high field. Several methine carbon signal appeared in the range of δ_C_ 50.1–58.4 and 72.1 (Figures S4 and S5). The ^1^H NMR spectrum showed δ_H_ 10.27 (1H, s), which was the peak of H-N of indole, and several amide N-H proton appeared in the range of δ_C_ 8.52, 8.44, 8.37, 8.25, 8.09, 8.04, 7.97. The ^1^H-^1^H COSY spectrum revealed the following proton-proton correlations: δ_H_ 0.88 (methyl of the n-decanoyl chain) with δ_H_ 1.25 (methylene of the n-decanoyl chain); δ_H_ 0.96 (methyl of MeGlu^12^) with δ_H_ 2.56 (β-H of MeGlu^12^); δ_H_ 6.54 with δ_H_ 7.15 and 7.63 (aromatic protons of Trp^1^); and δ_H_ 6.70 with δ_H_ 7.20 (aromatic protons of Kyn^13^) (Figure S6). The HSQC spectrum revealed correlations between the following proton and carbon signals: δ_H_ 0.88 (3H,t,J = 6.0 Hz) with δ_C_ 14.5 (methyl of the n-decanoyl chain); δ_H_ 0.96 (3H,d,J = 6.0 Hz) with δ_C_ 15.8 (methyl of MeGlu^12^); δ_H_ 1.22 (3H) with δ_C_ 17.2 (methyl of D-Ala^8^); and δ_H_ 1.21 (3H) with δ_C_ 32.6 (methyl of Thr^4^) (Figure S7). The HSQC spectrum showed that the a-methine protons of Orn^6^, D-Ala^8^, and Kyn^13^ correlated with carbon signals at δ_C_ 54.6, 51.28, and 50.1, respectively (δ_H_ 4.35, 4.27, and 5.03, respectively). The HSQC spectrum revealed the following proton-carbon correlations: δ_H_ 3.12/3.23 with δ_C_ 27.8 (β-methylene of Trp^1^); δ_H_ 5.40 with δ_C_ 72.1 (β-methine of Thr^4^); δ_H_ 1.85/1.76 with δ_C_ 29.2 (β-methylene of Trp^1^); δ_H_ 2.42/2.17 with δ_C_ 40.3 (β-methylene of MeGlu^12^); δ_H_ 3.85 with δ_C_ 63.2 (β-methylene of D-Ser^11^); and δ_H_ 3.57/3.47 with δ_C_ 41.9 (β-methylene of Kyn^13^). The HMBC experiment provided definitive evidence for the linkage between the proton at position δ_H_ 1.21 (methyl of Thr^4^) with the groups β-C (δ_C_ 71.9), methyl (δ_C_ 32.6), and a-C (δ_C_ 57.4) of Thr ^4^, as indicated by their long-range coupling. δ_H_ 7.54 with δ_C_ 110.0, 121.4, 128.4 and 138.0 of Trp^1^; δ_H_ 7.32 with δ_C_ 119.7 and 128.7, δ_H_ 7.20 with δ_C_ 153.1 and 132.5 of Kyn^13^. The HMBC experiment provided definitive evidence for the linkage between the Thr^4^methyl protons δH1.21,3H, d, J = 6.0 Hz and the β-carbon (δ_C_ 71.9), methyl carbon (δ_C_ 32.6), and a-carbon (δ_C_ 57.4) of the same residue, as indicated by their long-range correlations (Figure S8). Additionally, HMBC correlations were observed for the Trp^1^aromatic protons: δ_H_ 7.54 with δ_C_ 110.0, 121.4, 128.4, and 138.0; and for the Kyn^13^ aromatic protons: δ_H_ 7.32 with δ_C_ 119.7 and 128.7, and δ_H_ 7.20 with δ_C_ 132.5 and 153.1. The ^1^H NMR and ^13^C NMR data were very similar to those reported for daptomycin [25]^.^ The ^1^H and ^13^C NMR data for compound **1** were shown in Table S1. Daptomycin was a cyclic tridecapeptide that comprises two D-amino acids and several non-proteinogenic amino acids, including kynurenine (Kyn), ornithine (Orn), and L-3-methylglutamic acid (MeGlu). Its N-terminus was acylated with an n-decanoyl fatty acid chain, and its C-terminal carboxylate (from Kyn^13^) forms a lactone bond with the side-chain hydroxyl group of Thr^4^, giving rise to a cyclic decapeptide core.

The amino acid sequence is:

dec-^1^Trp-Asn-Asp-Thr-Gly-Orn-Asp-D-Ala-Asp-Gly-D-Ser-MeGlu-Kyn^13^.

Compound **1** is a structural analog of daptomycin.

### 3.3. Inhibitory Effect of Compound **1** on Hyphal Growth of Rhizoctonia solani

**Figure 2:**
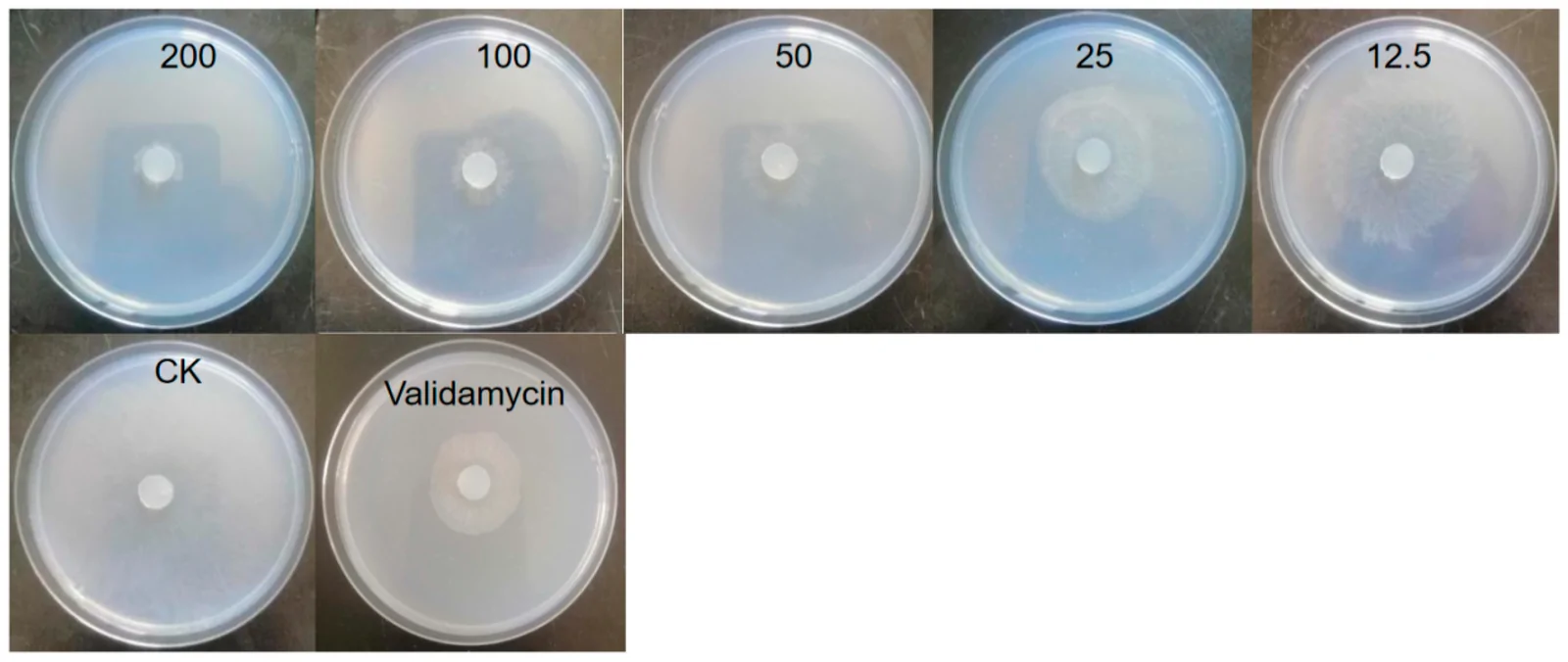
Inhibitory effects of compound **1** against *Rhizoctonia solani* (μg/mL). Note: CK is the control group. The concentration of validamycin is 100 μg/mL.

Table S2 clearly demonstrated that compound **1** significantly inhibited the mycelial growth of *R. solani* on agar plates relative to the untreated control (Figure 2). The antifungal activity was concentration-dependent; as the concentration of compound **1** increased, the colony diameter progressively decreased, and the inhibition rate rose accordingly. At a concentration of 200 μg/mL, compound **1** achieved a 93.07% inhibition rate, with a calculated IC_50_ value of 14.9 μg/mL. By contrast, the reference fungicide validamycin, tested at 100 μg/mL, produced only 72.56% inhibition rate. Furthermore, the colony diameter in the compound **1**-treated group exhibited a non-linear correlation with the applied concentration. Collectively, these findings suggested that compound **1** exerted a strong inhibitory effect against *R. solani* and outperforms validamycin under the tested conditions.

### 3.4. Whole-Genome Sequencing and Genomic Characterization of Streptomyces sp. F204

#### 3.4.1. Genome Sequencing and Assembly of *Streptomyces* sp. F204

The preliminary assembly contained two contigs with a total length of approximately 8.744 Mb, including a major chromosomal contig, ctg_1, of 8,273,549 bp and a second unclassified contig, ctg_2, of 470,178 bp. PlasFlow classified ctg_1 as chromosome, whereas ctg_2 remained unclassified and was not annotated as a plasmid. Therefore, the chromosomal sequence of *Streptomyces* sp. F204 was represented by a non-circular chromosomal contig of 8, 273, 549 bp, with a GC content of 71.11%. The assembly showed high completeness, with 99.4% complete BUSCOs. The structure and nucleotide-level features of the assembled genome of *Streptomyces* sp. F204 were analyzed using Prokka software. A total of 8022 genes were predicted, and the coding density of the genome was 86.72% (including pseudogenes). Among these, 7865 protein-coding sequences (CDSs) were identified, representing 86.24% of the genome. Predicted non-coding RNAs included 84 tRNA genes, one tmRNA gene, 18 rRNA genes, and 54 miscRNA (miscellaneous RNA, i.e., other unknown RNA) genes. The detailed results of coding gene prediction were presented in Table S3.

**Figure 3:**
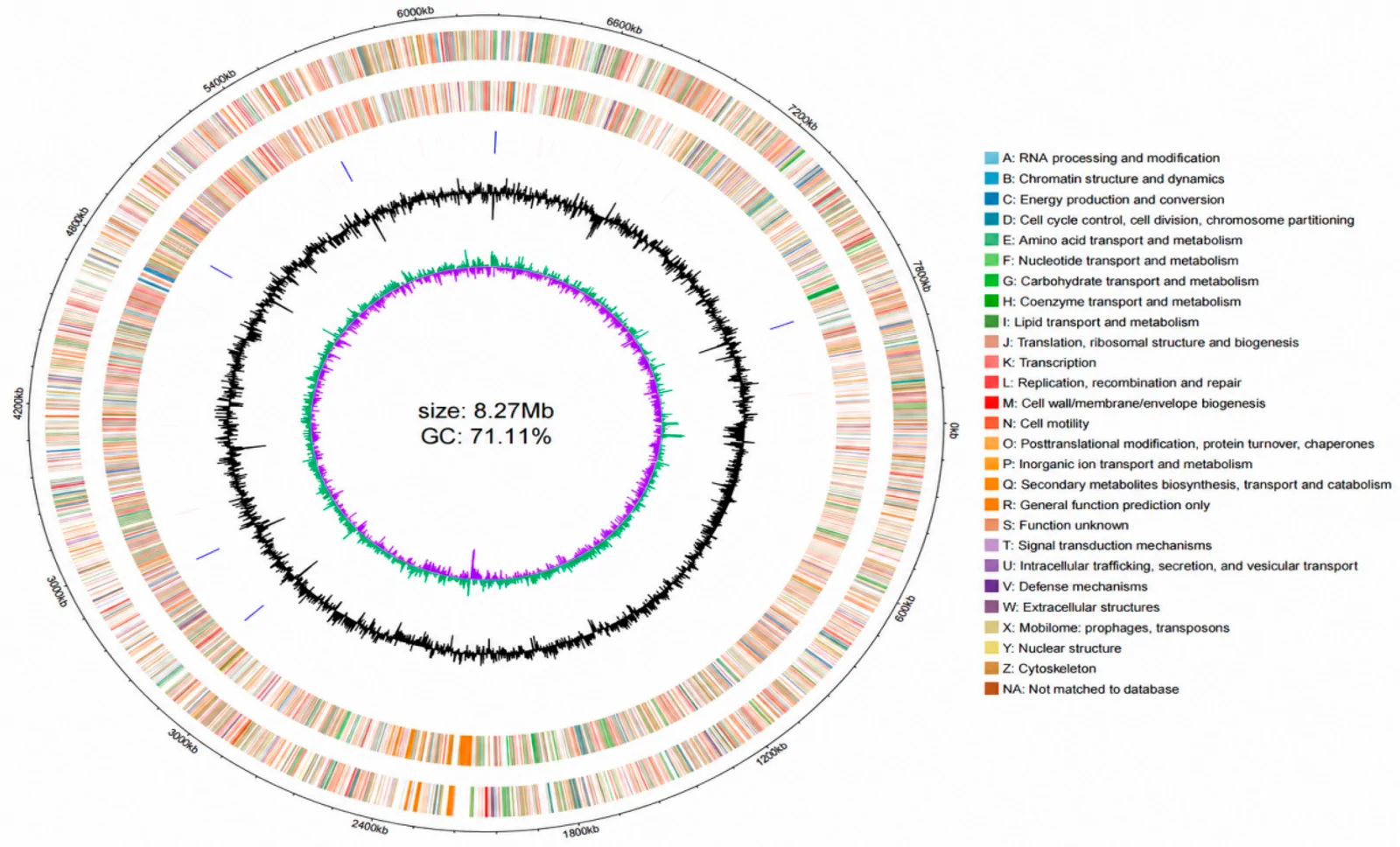
Genomic circle diagram of *Streptomyces* sp. F204, from outside to inside: first circle, genomic coordinates; in the second circle, genes on the positive strand of the genome sequence; different colors represent different COG functional classifications; in the third circle, genes on the negative strand of the genome sequence; different colors represent different COG functional classifications; in the fourth circle, rRNA and tRNA on the genome sequence; rRNA is blue, tRNA is red; fifth, the GC content of the genome sequence; in the sixth circle, the GC skew of the genome sequence; green indicates that the G content is greater than the C content, and purple indicates that the G content is less than the C content.

Using the R package circlize, the predicted genomic information of *Streptomyces* sp. F204—including genome structural annotation, GC distribution, and COG functional annotation—was integrated to generate a circular genomic map. Circlize facilitates genome visualization, enabling clearer exploration of relationships among genomic components or positions, as shown in Figure 3 (Circular genomic map of *Streptomyces* sp. F204). The GC skew pattern was consistent with the typical bacterial genomic architecture, where a bias between the leading and lagging strands was observed.

In addition, the closest phylogenetic strains were selected to calculate the ANI and dDDH. The ANI values of the model strains *Streptomyces puniciscabiei* S77 (GCF_006715785) and *Streptomyces fodineus* TW1S1 (NZ_CP017248), which were closely related to F204, are 91.64% and 90.43%, respectively, while the dDDH values were 45.00% and 41.40%, respectively. The standard threshold values for discriminating new bacterial species based on low ANI and dDDH were 95% and 70% (Table S8).

#### 3.4.2. Functional Annotation of the *Streptomyces* sp. F204 Genome

**Figure 4:**
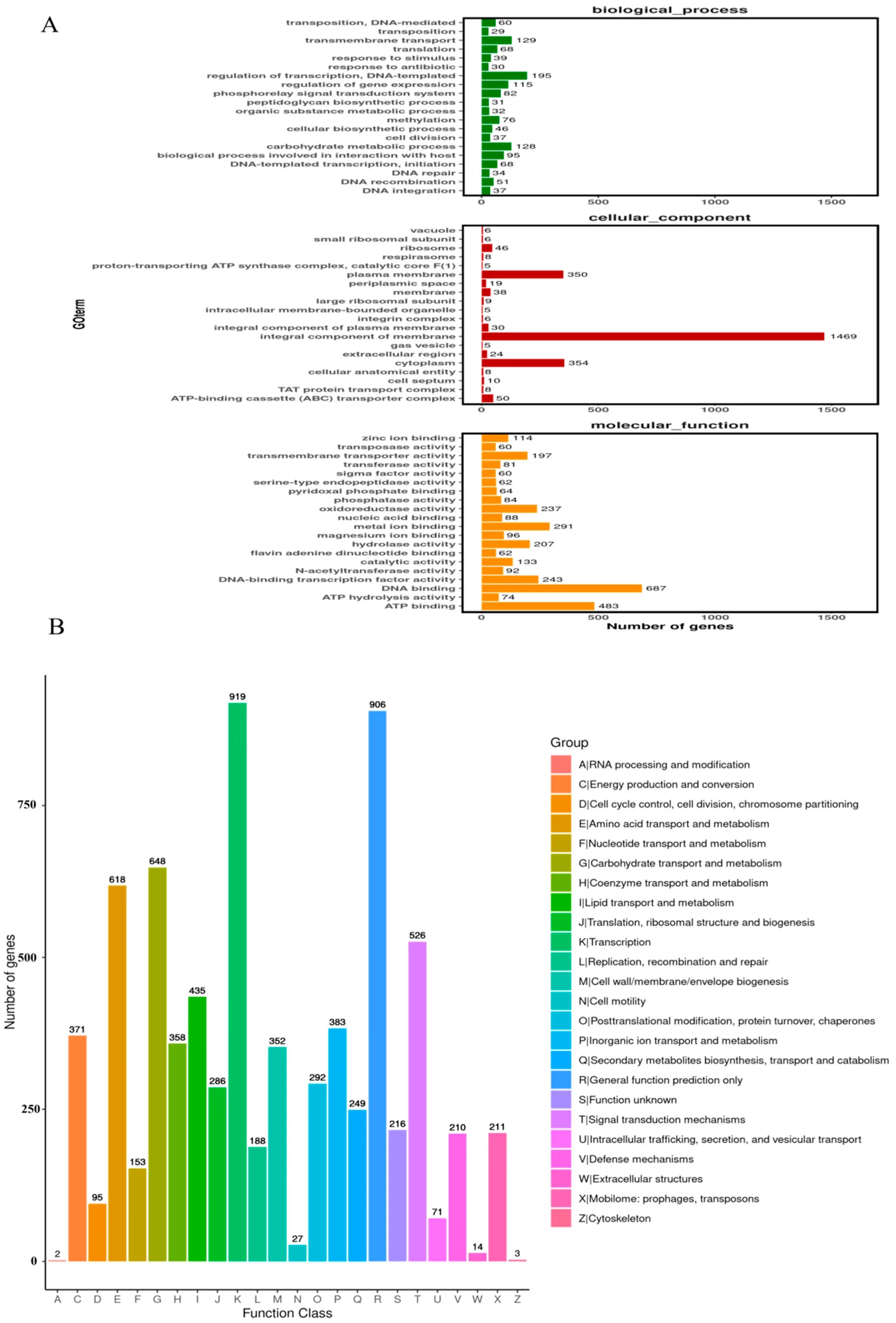
Genomic features and functional annotation. (**A**) Distribution of *Streptomyces* sp. F204 GO functional annotations. (**B**) Distribution of functional annotations of *Streptomyces* sp. F204 COG.

Functional annotation of the *Streptomyces* sp. F204 genome revealed a broad genetic basis for metabolism, environmental adaptation, and secondary metabolite production. GO annotation assigned functions to 3331 genes (Figure 4A). In the molecular function category, DNA binding and ATP-related functions were among the most enriched terms, suggesting active transcriptional regulation and energy-dependent cellular processes. In the cellular component category, abundant genes were associated with membrane components and the plasma membrane, indicating a substantial capacity for membrane transport, environmental sensing, and interaction with extracellular substrates.

COG classification further supported the metabolic versatility of strain F204 (Figure 4B). A total of 7533 genes were assigned to 26 functional categories. The most represented categories included transcription (919 genes), amino acid transport and metabolism (618), carbohydrate transport and metabolism (548), signal transduction mechanisms (526), lipid transport and metabolism (435), inorganic ion transport and metabolism (383), energy production and conversion (371), coenzyme transport and metabolism (358), and cell wall/membrane/envelope biogenesis (352). These distributions indicated that F204 possessed extensive capacities for nutrient utilization, regulatory control, energy generation, and adaptation to changing environmental conditions. Notably, 249 genes were assigned to secondary metabolite biosynthesis, transport, and catabolism, highlighting the potential of this strain to produce bioactive natural products. In addition, 906 genes were annotated as encoding proteins of unknown function, suggesting that F204 might harbor unexplored biosynthetic or adaptive functions.

KEGG pathway annotation identified 3532 protein-coding genes associated with metabolic and cellular pathways (Figure S9). Among the five major KEGG categories, Metabolism contained the largest number of annotated genes, followed by Environmental Information Processing. This pattern indicated that *Streptomyces* sp. F204 had a well-developed metabolic network and strong capacity to respond to external environmental stimuli. Together, these functional profiles indicated that *Streptomyces* sp. F204 combined metabolic versatility with strong regulatory and environmental-response capacities, providing a genomic basis for its potential production of diverse and possibly novel secondary metabolites.

In this study, the HMMER model was used to perform CAZy functional annotation on the whole genome of *Streptomyces* sp. F204. The genome of this strain was found to encode multiple types of carbohydrate-metabolism-related functional components, including 115 glycoside hydrolases (GHs), 66 glycosyltransferases (GTs), 7 polysaccharide lyases (PLs), 17 carbohydrate esterases (CEs), 22 auxiliary activity redox enzymes (AAs), and 39 carbohydrate-binding modules (CBMs). Notably, the biosynthetic potential of GHs and GTs was particularly prominent (115 and 66 genes, respectively), and these two enzyme families possessed catalytic functions for synthesizing glycoside-type active products (Figure S10). This finding not only reveals the significant advantages of this strain in the biosynthetic pathways of glycoside antibiotics but also provides an important molecular biological basis for subsequent targeted strain improvement and the development of secondary metabolites.

#### 3.4.3. AntiSMASH-Based Prediction Analysis of Secondary Metabolites in *Streptomyces* sp. F204

The whole-genome sequencing results of *Streptomyces* sp. F204 were submitted to antiSMASH 6.0.0, revealing that the genome contains 23 BGCs for secondary metabolites (Table S4). These included one cluster each for ranthipeptides, thioamides, T2PKS, T1PKS, redox-cofactors, phenazines, ectoine, and NAPAA; four RiPP-like clusters; three terpene clusters; two T3PKS clusters; three siderophore clusters; and three additional hybrid clusters. Among these, clusters 1.15, 1.18, and 2.3 shared 100% similarity with known compound biosynthetic gene clusters, indicating a high probability that the strain produces the corresponding secondary metabolites. The biosynthetic gene cluster showed 100% similarity to the corresponding reference cluster, indicating the potential capacity of strain F204 to produce the related metabolite. Specifically, cluster 1.15 is a terpene cluster. Cluster 1.18 is an ectoine cluster. Cluster 2.3 is a siderophore cluster. Clusters 1.1, 1.2, 1.12, 1.19, and 2.2 show very low similarity to known biosynthetic gene clusters, and antiSMASH did not provide reference predictions for them. Nevertheless, among these, cluster 1.2 (thioamides) is noteworthy; thioamides possess high biological activities, including anti-inflammatory, bactericidal, and anti-tuberculosis effects. Cluster 1.19 non-α polyamino acids (NAPAA), such as ε-poly-L-lysine), represents an important compound class in newly discovered antibiotics. These findings suggested that such gene clusters might produce natural active antibacterial agents with structures and biosynthetic mechanisms distinct from known secondary metabolites. Clusters 1.5, 1.6, 1.7, and 1.17 show relatively high similarity (>50%) to known gene clusters, also indicating a strong likelihood of producing such secondary metabolites, this requires functional validation. Among clusters with less than 50% similarity, there are also numerous antibiotic-related gene clusters. For example, cluster 1.13 (thiopeptide, LAP class azole-containing compounds) had antibacterial activity, and cluster 1.16 (phenazine) was primarily used in medicine, as an organic synthesis intermediate, and in biochemical research. Low-similarity biosynthetic gene clusters might represent potential sources of structurally diverse or previously uncharacterized secondary metabolites, which warrant further experimental investigation to determine their chemical structures and biological activities.

### 3.5. Identification of Discriminatory Metabolites in Response to Streptomyces sp. F204 in Different Media

**Figure 5:**
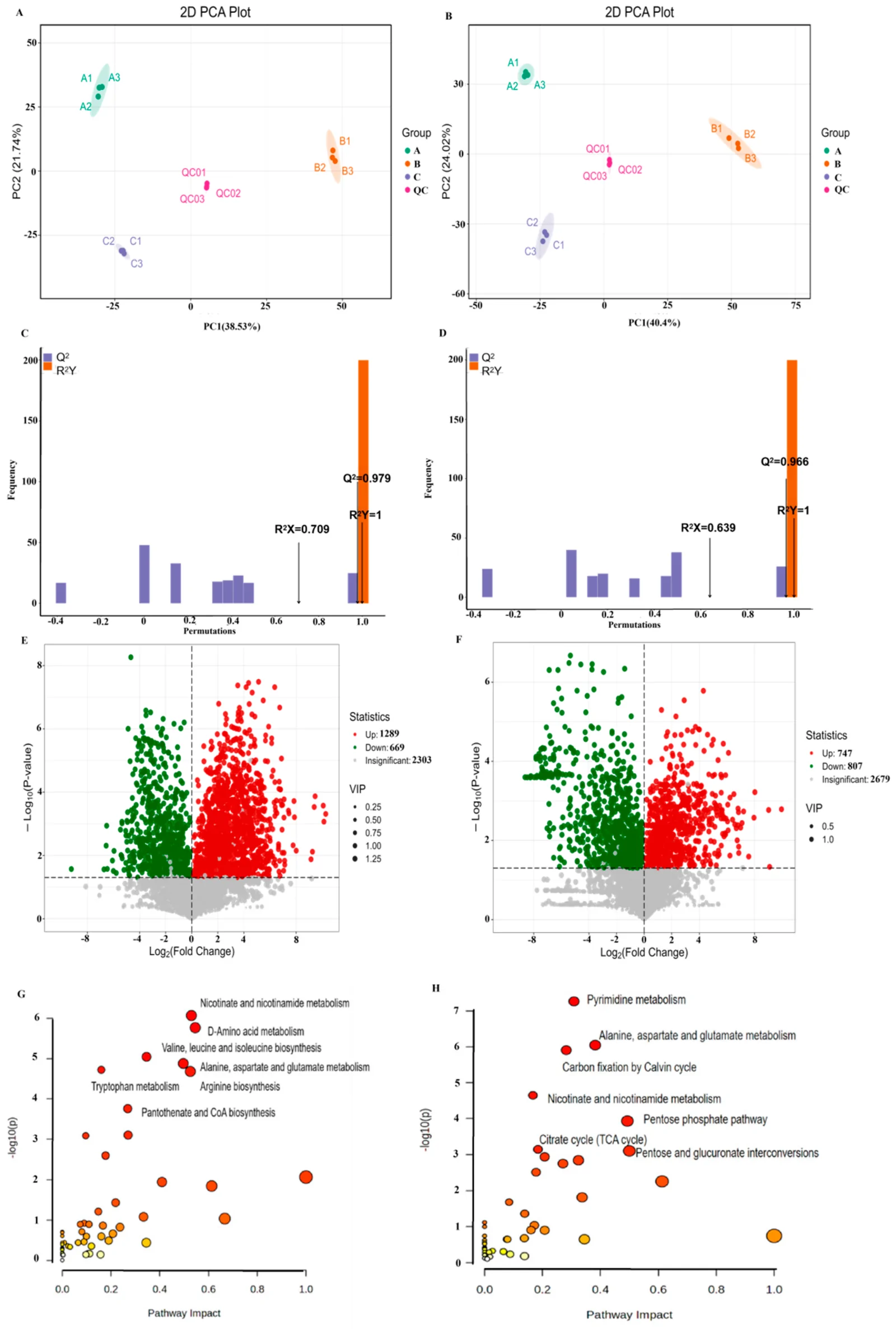
Metabolic profiling and KEGG topological analysis map. (**A**) PCA score diagram (cation). (**B**) PCA score diagram (anion). (**C**) OPLS-DA score plot in B vs. A. (**D**) OPLS-DA score plot in C vs. A. (**E**) Volcano map in B vs. A. (**F**) Volcano map in C vs. A. (**G**) Pathway enrichment analysis of B vs. A. (**H**) Pathway enrichment analysis of C vs. A. n = 3 per group.

Metabolic changes were assessed using principal component analysis (PCA), as shown in Figure 5A,B, for both positive and negative ion modes. On the *X*-axis, PC1 accounted for 38.53% (positive ion mode) and 40.4% (negative ion mode) of the data variability. The *Y*-axis represented PC2, explaining 21.74% of the variability in positive ion mode and 24.02% in negative ion mode. The plot illustrated the distribution of nine sample groups based on PC1 and PC2. The left side of the plot was predominantly occupied by groups Gause’s Synthetic medium (A) and ISP4 medium (C), while the right side was mainly occupied by group Medium No. 2 (B). This distribution indicated significant differences in sample characteristics among groups A, C, and B.

Metabolomic analysis was further performed using orthogonal partial least squares discriminant analysis (OPLS-DA), which revealed a distinct separation between the metabolic profiles of each pair of groups. The OPLS-DA model demonstrated high interpretability and predictive accuracy, with R^2^Y values of 1 and Q^2^ values of 0.979 for the comparison of B vs. A, and R^2^Y values of 1 and Q^2^ values of 0.966 for C vs. A, as shown in Figure 5C,D. These results confirmed the reliability of the OPLS-DA model without overfitting, underscoring the significant impact of different culture media on the metabolic processes of *Streptomyces* sp. F204, leading to discernible changes in its metabolite composition.

The volcano plot visually illustrates significant differences in metabolite levels between two sample groups. For the B vs. A comparison, 669 downregulated metabolites (green dots) and 1289 upregulated metabolites (red dots) are shown. For the C vs. A comparison, 807 downregulated and 747 upregulated metabolites were indicated. Metabolites with no significant difference were depicted in gray (Figure 5E,F). This visualization aided in discerning the magnitude and statistical significance of differences in metabolite levels between each pair of groups.

*Streptomyces* sp. F204 was grown in Gause’s synthetic medium (A), Medium No. 2 (B), and ISP4 medium (C). Metabolomic profiling identified 896 differential metabolites between B and A and 492 between C and A. The inter-group relationships, visualized as Venn diagrams in Figure S11, demonstrated that different OSMAC culture conditions produce distinct secondary metabolite profiles. For the B vs. A comparison, pathway topology analysis identified 12 metabolic pathways with significant differences (*p* < 0.05, Impact > 0), including: nicotinate and nicotinamide metabolism; D-amino acid metabolism; valine, leucine, and isoleucine biosynthesis; alanine, aspartate, and glutamate metabolism; tryptophan metabolism; arginine biosynthesis; pantothenate and CoA biosynthesis; phenylalanine, tyrosine, and tryptophan biosynthesis; monobactam biosynthesis; galactose metabolism; novobiocin biosynthesis; beta-alanine metabolism, as clearly shown in Figure 5G. Pathways positioned closer to the top and right are those with greater impact and higher significance. Notably, “nicotinate and nicotinamide metabolism” and “D-amino acid metabolism” were among the most affected. A total of 82 metabolites, including 1-methylnicotinamide, nicotinic acid, and nicotinamide mononucleotide, were implicated in these pathways (Table S5).

Similarly, for the C vs. A comparison, pathway topology analysis revealed 15 metabolic pathways with significant differences (*p* < 0.05, Impact > 0): pyrimidine metabolism; alanine, aspartate, and glutamate metabolism; carbon fixation by the Calvin cycle; nicotinate and nicotinamide metabolism; pentose phosphate pathway; citrate cycle (TCA cycle); pentose and glucuronate interconversions; other carbon fixation pathways; starch and sucrose metabolism; pantothenate and CoA biosynthesis; galactose metabolism; beta-alanine metabolism; glyoxylate and dicarboxylate metabolism; butanoate metabolism; and methane metabolism (Figure 5H). A total of 68 metabolites, such as glutamine, citric acid, and succinic acid semialdehyde, were implicated in these pathways (Table S6).

**Figure 6:**
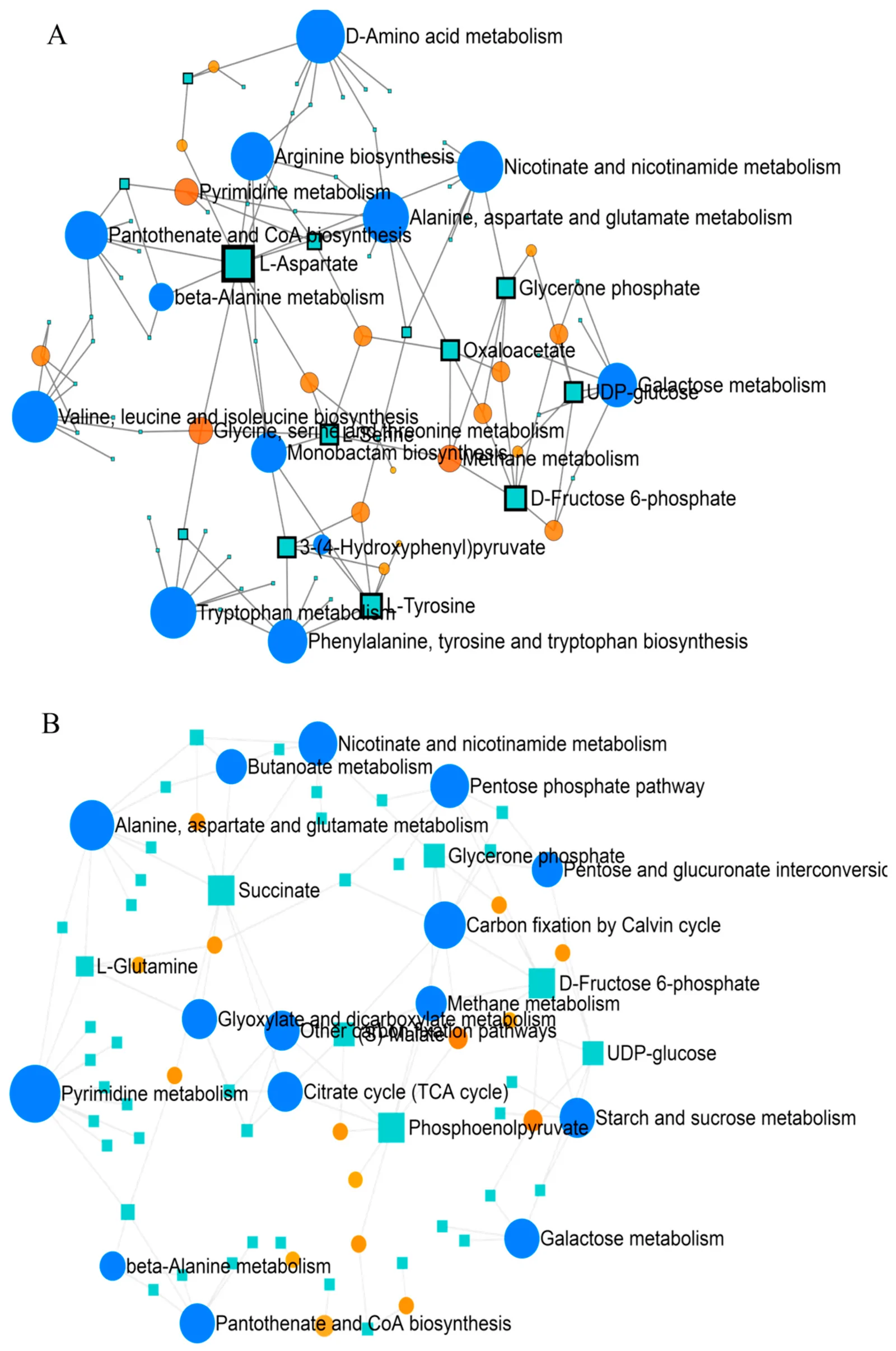
The KEGG global metabolic network: (**A**) B vs. A; (**B**) C vs. A. Note: Blue circles typically representing major metabolic pathways, such as those corresponding to nicotinate and nicotinamide metabolism and D-amino acid metabolism, are displayed at a larger size, indicating their prominence. Green squares represent metabolites, while the connecting lines represent metabolic reactions or chemical relationships between these compounds.

Figure 6A,B illustrated the KEGG global metabolic network generated using the Network Explorer analysis module. In the figure, major metabolic pathways were indicated by blue circles, which were displayed at a larger size to denote their importance. Examples included pathways related to nicotinate and nicotinamide metabolism as well as D-amino acid metabolism. Green square represent metabolites, while the connecting lines represent metabolic reactions or chemical relationships between these compounds. Several compounds associated with nicotinate and nicotinamide metabolism—including nicotinic acid, nicotinamide mononucleotide, succinic acid semialdehyde, nicotinic acid adenine dinucleotide, nicotinate mononucleotide, maleic acid, and L-aspartic acid—are marked in green. Additionally, other compounds such as L-aspartic acid, L-alanine, D-alanine, D-ornithine, L-proline, and cis-4-hydroxy-D-proline are also marked in green; these are linked to D-amino acid metabolism, possibly indicating their greater importance or relevance within this specific network.

## 4. Discussion

The 23 BGCs identified in the genome of *Streptomyces* sp. F204 spanned diverse classes, including terpenes, T1PKS, T2PKS, T3PKS, phenazines, siderophores, ectoine, thioamides, RiPP-like peptides, and NAPAA. This diversity indicated the considerable biosynthetic potential of the genus *Streptomyces* for producing a wide array of secondary metabolites. Systematic analysis of these BGCs indicated that *Streptomyces* sp. F204 possessed a rich capacity for natural product biosynthesis. Notably, the strain contained not only BGCs with high homology to known antibiotics but also multiple clusters exhibiting very low similarity to any known counterparts, thereby providing a genetic foundation for the discovery of novel bioactive compounds.

In this study, cluster 1.15 (a terpene cluster) shared 100% similarity with a known biosynthetic gene cluster (BGC). Previous research has demonstrated that endophytic *Streptomyces* can produce over 150 compounds, including terpenes, most of which exhibit significant antibacterial activity [26,27]. Beyond their antimicrobial effects, terpenes possess various physiological activities, such as anti-inflammatory, analgesic, and anthelmintic properties, granting them dual potential in agricultural disease control and pharmaceutical development. The predicted clusters represent only biosynthetic potential, and that functional expression and bioactivity require experimental confirmation.

Cluster 1.18 (an ectoine cluster) also showed 100% similarity to a known BGC. Ectoine not only exhibits excellent skin repair and protective properties but also stabilizes biomacromolecules under extreme conditions, conferring a survival advantage for *Streptomyces* sp. F204 in environmental adaptation [28]. Although ectoine is widely used in high-end cosmetics and bioengineering products, its roles in microbial tolerance regulation and stress response have garnered increasing attention in recent years, suggesting that this metabolite may also participate in the secondary metabolic regulatory network of the strain under different culture conditions. This function is inferred from the literature and has not been verified in our strain.

Cluster 2.3, a highly conserved siderophore cluster, deserves particular attention. Siderophores are low-molecular-weight iron chelators secreted by microorganisms under iron-deficient conditions. They can act as competitive inhibitors of pathogen iron uptake systems and, through a “Trojan horse” strategy, be conjugated to antibiotics to overcome bacterial resistance [29]. Studies have confirmed that *Streptomyces* are a rich source of siderophores, which display diverse chemical structures and broad-spectrum antipathogenic activity [30]. Therefore, Cluster 2.3 might offer some insight into strategies for developing F204 as a novel antibiotic.

Several low-similarity gene clusters identified in this study are particularly noteworthy. Although cluster 1.2 (thioamides) shares very low similarity with known clusters, thioamide compounds are generally recognized for their potent anti-inflammatory, bactericidal, and anti-tuberculosis activities. Likewise, cluster 1.19 (NAPAA, ε-poly-L-lysine type) represents another highlight. NAPAA (non-α polyamino acids) have been confirmed to occur in the genomes of diverse microorganisms and are regarded as highly promising novel scaffolds for antibiotic development [31]. For instance, ε-poly-L-lysine, a natural antimicrobial agent, has been widely used in food preservation, and its derivatives have shown encouraging prospects in new drug discovery. NAPAA BGCs of microbial origin have also been reported in *Streptomyces* and are thought to produce antibacterial compounds such as stenothricin [32]. Furthermore, although clusters 1.13 (thiopeptide/LAP class) and 1.16 (phenazine) share less than 50% similarity with known clusters, they may be associated with antibiotic production, but this requires functional validation.

In this study, metabolomic analysis (PCA and OPLS-DA) confirmed that different culture media significantly influenced the metabolite profile of strain F204. This finding aligns with the typical regulatory characteristics of the “One Strain Many Compounds” (OSMAC) strategy in *Streptomyces*—namely, altering the culture environment to activate silent gene clusters enables the same strain to produce different types of secondary metabolites [33]. Our experimental results also supported this finding. Untargeted metabolomics is widely used to discover bioactive compounds from *Streptomyces*. For example, using this approach, new antibacterial compounds such as arylomycin were discovered in *Streptomyces parvulus* HCCB10043 [34]. This phenomenon is common in *Streptomyces*: when *Streptomyces aurantiacus* MH191 was grown on six different culture media, a total of 205 putative compounds were detected, among which antifungal metabolites such as manumycin family members and undecylprodigiosin were produced only in specific media [35]. Thus, the secondary metabolite production of *Streptomyces* sp. F204 exhibits significant culture-condition dependency, necessitating the adoption of diversified fermentation strategies in subsequent work on the isolation and identification of bioactive substances.

Pathway enrichment analysis reveals that the nicotinate and nicotinamide metabolism pathway is significantly enriched in both the B vs. A and C vs. A comparisons (*p* < 0.05). The differential expression of this pathway warrants further investigation. Nicotinate mononucleotide is a key intermediate in NAD^+^ biosynthesis, which may influence cellular redox balance. It is well established that nicotinamide adenine dinucleotide (NAD^+^) is a core coenzyme involved in cellular redox reactions, energy metabolism, and signal transduction, and that the nicotinate and nicotinamide metabolism pathway plays an important role in regulating antibiotic susceptibility and resistance. In multidrug-resistant *Mycobacterium tuberculosis*, perturbation of this pathway is closely associated with the resistant phenotype [36]. Therefore, nicotinate/nicotinamide metabolism may participate in precursor supply for secondary metabolites or in regulatory signal transduction.

The D-amino acid metabolism pathway also exhibited significant differences in the B vs. A comparison, while enrichment of the alanine, aspartate, and glutamate metabolism pathway was observed in the C vs. A comparison. D-amino acids are known not only to act as unique bacterial metabolic signaling molecules but also to participate in processes such as quorum sensing and biofilm formation [37]. Alterations in these pathways may reflect differences in the availability of amino acid precursors in strain F204 under different culture conditions, thereby affecting the biosynthesis efficiency of non-ribosomal peptide synthetase (NRPS) products that depend on amino acids. L-aspartic acid and L-phenylalanine are precursors for multiple secondary metabolites; methionine is involved in sulfur metabolism and methylation reactions; and maleic acid is a TCA cycle-related organic acid. Their differential accumulation suggests that the strain’s primary and secondary metabolic networks are modulated under the tested conditions, potentially affecting the production of bioactive compounds.

Integrating genomic and metabolomic results, *Streptomyces* sp. F204 possessed both highly homologous known clusters (providing direct pathways for efficient synthesis of known antimicrobial compounds) and low-similarity unknown clusters (offering a genetic foundation for the synthesis of novel scaffolds). This characteristic particularly highlighted the dual advantage of *Streptomyces* as a “natural drug synthesis factory.” Recent studies have shown that the genomes of *Streptomyces* from extreme environments typically contain a large number of uncharacterized BGCs, providing new molecular entity sources to combat antibiotic resistance [38].

Nevertheless, this study had certain limitations. Although antiSMASH analysis predicted abundant BGCs, most of the differential metabolites detected by metabolomics were not yet fully identified. The lack of structure elucidation work, such as NMR and LC–MS/MS, restricts the accurate assignment of specific active compounds. Moreover, the direct physiological significance of the observed changes in the nicotinate/nicotinamide metabolism pathway still requires validation through gene knockout and complementation experiments. In future work, we will optimize the culture parameters of *Streptomyces* sp. F204 based on the OSMAC strategy, combined with genome-mining-guided fractionation techniques, to perform targeted isolation and bioactivity evaluation of novel metabolites corresponding to low-similarity BGCs, thereby fully exploiting the potential of this strain in the biocontrol of rice sheath blight and the discovery of new antibiotics.

## 5. Conclusions

In conclusion, *Streptomyces* sp. F204 shows strong potential as a biocontrol resource against *R. solani*. Bioassay-guided isolation identified compound **1** as a daptomycin-like metabolite with notable antifungal activity, indicating that this strain could produce effective inhibitory substances. Genome sequencing revealed 23 predicted secondary-metabolite biosynthetic gene clusters, including clusters similar to known bioactive products and clusters with low similarity to characterized pathways, suggesting capacity for both known and potentially novel metabolites. Metabolomic analysis further showed that different culture media substantially altered metabolite production, supporting the usefulness of the OSMAC strategy for activating secondary metabolism in F204. Overall, the combined chemical, genomic, and metabolomic evidence indicated that *Streptomyces* sp. F204 was a valuable source of antifungal natural products. Further purification of active metabolites, functional analysis of biosynthetic clusters, and greenhouse or field evaluation will help clarify its practical field value for rice sheath blight control and future sustainable agricultural disease management applications. We have also emphasized the need for future experimental validation of the metabolomic predictions and the potential application of strain F204 in biocontrol strategies.

## Data Availability

The original contributions presented in this study are included in the article. Further inquiries can be directed to the corresponding authors. The whole-genome assembly of *Streptomyces* sp. strain F204 has been deposited in the European Nucleotide Archive under accession GCA_988916585.

## Supplementary Materials

The following supporting information can be downloaded at: https://www.mdpi.com/article/10.3390/jof12100782/s1, Figure S1: Mass spectrum of compound **1**; Figure S2: ^1^H NMR spectrum of compound **1**; Figure S3: ^13^C NMR spectrum of compound **1**; Figure S4: DEPT-90° spectrum of compound **1**; Figure S5: DEPT-135° spectrum of compound **1**; Figure S6: ^1^H-^1^H COSY spectrum of compound **1**; Figure S7: HSQC spectrum of compound **1**; Figure S8: HMBC spectrum of compound **1**; Figure S9: Distribution of *Streptomyces* sp. F204 KEGG functional annotations; Figure S10: A pie chart illustrates the CAZymes encoded; Figure S11: Venn diagram of differential metabolites among groups. Table S1: ^1^H and ^13^C chemical shifts of the assigned residues of compound **1** respectively(CD_3_OD); Table S2: Inhibitory effects of compound **1** on *Rhizoctonia solani*; Table S3: Prediction results of the gene encoding *Streptomyces* F204; Table S4: Analysis of potential secondary metabolic biosynthetic gene clusters in *Streptomyces* sp.; Table S5: Identification of differential metabolites (B vs. A); Table S6: Identification of differential metabolites (C vs. A); Table S7: Mobile phase gradient program for the T3 column; Table S8: Values of ANI and dDDH between F204 and its closest species.
