# Supplementary material for: Antifungal Activity of Streptomyces sp. F204 Against Rhizoctonia Solani and Whole-Genome Sequencing and Metabolomic Analysis
Source: J Fungi (Basel). 2026 Oct 19;12(10):782. doi: 10.3390/jof12100782 (PMC13705709; doi:10.3390/jof12100782)
Supplement: Supplementary file 1 [file jof-12-00782-s001.zip › jof-4453347-supplementary.pdf]

Supplementary material

Antifungal activity of Streptomyces sp. F204 against Rhizoctonia solani and whole-genome sequencing and metabolomic

Long Hui Shen[1] , Jin Yan Xue[2], Lin Yan Zhu[1*]

[1] Department of Pharmacy, The Affiliated Women and Children's Hospital of Ningbo University, Ningbo, Zhejiang, 315000, China.

ORCID: 0009-0006-4949-0831

[2] School of Pharmacy, Liaoning University of Traditional Chinese Medicine, 77 Shenming Road, Dalian, Liaoning, 116600, China.

ORCID :0009-0001-6160-1737

Department of Pharmacy, The Affiliated Women and Children's Hospital of Ningbo University, Ningbo, Zhejiang, 315000, China.

Tel: +86-87083401. E-mail: fezhulinyan@nbu.edu.cn

ORCID :0009-0001-7471-3455

Table S1. ^{1}H and ^{13}C chemical shifts of the assigned residues of compound 1 respectively(CD_3OD)

Residue	$CH_α$	$CH_β$	Aromatics
Trp 1	4.60	3.12,3.23, (27.8)	7.15, 7.63, 7.06, 7.54, 6.97, 10.27 (indole HN)
Asn 2	4.52	2.15,2.40	
Asp3	4.75	2.28, 2.15	
Thr 4	4.79	5.40, (72.1)	
Gly 5	3.97		
Orn 6	4.35, (54.6)	1.85, 1.76, (29.2)	
Asp 7	4.52	2.39, 2.15	
D-Ala8	4.27, (51.28)	1.22, 17.2	
Asp9	4.65	2.75, 2.60	
Gly 10	3.94	—	
D-Ser 11	4.58	3.85, 63.2	
MeGlu 12	4.57	2.42, 2.17, (40.3)	
Kyn 13	5.03, (50.1)	3.57,3.47, (41.9)	7.32, 7.20, 6.52, 6.70

Note: The values reported are based on the data shown in Fig. S2-7, ^{1}H, ^{13}C, and HSQC spectrum

Table S2. Inhibitory effects of compound 1 on *Rhizoctonia solani.*

Concentration (μg/mL)	Diameter (mm)			Average diameter (mm)	Inhibition ratio (%)
	1	2	3		
200	15.12	15.42	16.07	15.53±0.39	93.07±0.49
100	18.01	18.16	18.52	18.23±0.21	89.71±0.26
50	25.28	25.33	26.02	26.21±0.69	79.73±0.86
25	37.24	36.67	35.84	36.58±0.57	66.77±0.71
12.5	54.06	54.15	55.14	54.45±0.48	44.43±0.61
CK	90	90	90	90.00±0.00	0
Validamycin	31.62	31.82	32.44	31.95±0.35	72.56±0.44

Note: CK is the control group. The concentration of validamycin is 100 μg/mL. The results of Duncan's multiple-interval test showed that at the significance level of p <0.05.

Table S3. Prediction results of the gene encoding *Streptomyces* F204

Type	Number	Total_len	Average_len	Percentage of genome (%)
Gene	8,022	7,582,410	945	86.72
CDS	7,865	7,540,416	959	86.24
tRNA	84	6,597	79	0.08
23S rRNA	6	18,663	3,110	0.21
16S rRNA	6	9,136	1,523	0.10
5S rRNA	6	663	110	0.01
tmRNA	1	369	369	0.00
miscRNA	54	6,566	122	0.08

Table S4. Analysis of potential secondary metabolic biosynthetic gene clusters in *Streptomyces* sp. F204

Region	Type	From	To	Most similar known cluster	Similarity
1.1	ranthipeptide	657,422	682,877	-	-
1.2	thioamides	1,012,459	1,034,943	-	-
1.3	RiPP-like	1,318,7961	1,326,856	informatipeptides	42%
1.4	T2PKS	1,767,670	1,840,152	Hiroshidine	27%
1.5	terpene	1,881,928	1,906,370	hope	92%
1.6	T1PKS	2,104,218	2,222,372	Heronamide A / heronamide B / heronamide C / heronamide D / heronamide E / heronamide F	62%
1.7	NRPS, arylpolyene, ladderane, other, T1PKS	2,257,396	2,370,423	Pepticin A	90%
1.8	redox-cofacto r	2,587,894	2,609,988	kanamycin	2%
1.9	T3PKS	2,868,496	2,908,142	daptomycin	6%
1.10	T3PKS	2,959,741	3,000,802	herboxidiene	8%
1.11	T1PKS, hglE-KS	3,053,440	3,105.275	cinnamycin	19%
1.12	RiPP-like	3,253,002	3,264,333	-	-
1.13	thiopeptide , LAP	3,274,841	3,301,987	goadsporin	12%
1.14	siderophore	3,421,996	3,431,379	-	-
1.15	terpene	4,095,101	4,116,024	albaflavenone	100%
1.16	phenazine	6,374,567	6,395,055	lomofungin	39%
1.17	siderophore	6,528,007	6,539,779	desferrioxamineB/desferrio xamineE	83%
1.18	ectoine	7,708,031	7,718,435	ectoine	100%
1.19	NAPAA	7,955,613	7,989,488	-	-
2.1	RiPP-like	20,012	30,907	Lipopeptide 8D1-1/lipopeptide 8D1-2	4%
2.2	RiPP-like	108,546	119,862	-	-
2.3	terpene	140,358	162,520	geosmin	100%
2.4	siderophore	314,118	327,232	grincamycin	5%

Table S5. Identification of differential metabolites (B vs. A)

No.	Metabolite	Formula	Retention time (min)	Precursor (g/mol)	Mass (g/mol)	Trend
1	1-Methylnicotinamide	$C_7H_9N_2O$	1.412	137.0672	137.0715	down
2	Nicotinic acid	$C_6H_5NO_2$	0.6584	123.0398	123.0320	down
3	Nicotinamide mononucleotide	$C_{11}H_{15}N_2O_8P$	3.2027	335.0647	334.0566	down
4	Succinic acid semialdehyde	$C_4H_6O_3$	1.1337	140.9935	102.0317	down
5	Nicotinic acid adenine dinucleotide	$C_{21}H_{27}N_6O_{15}P_2$	1.2263	664.1042	665.1010	down
6	Nicotinate mononucleotide	$C_{11}H_{15}NO_9P$	0.6809	300.0209	336.0484	up
7	1-Methylpyrrolinium	$C_5H_{10}N$	0.6089	129.0517	84.0813	down
8	L-Aspartic acid	$C_4H_7NO_4$	1.0529	134.0436	133.0375	up
9	Maleic acid	$C_4H_4O_4$	1.2103	115.0035	116.0110	up
10	1,4-Dihydro-1-methyl-4-oxo-3-pyridinecarboxamide	$C_7H_8N_2O_2$	4.5106	151.0611	152.0586	down
11	Dihydroxyacetone phosphate	$C_3H_7O_6P$	1.1284	229.0135	169.998	down
12	L-Phenylalanine	$C_9H_{11}NO_2$	2.0538	166.0690	165.079	up
13	L-Lysine	$C_6H14N_2O_2$	0.6459	147.1256	146.1055	up
14	Methionine	$C_5H_{11}NO_2S$	1.214	150.0573	149.0510	up
15	Glutamine	$C_5H_{10}N_2O_3$	0.9171	147.0751	146.0691	down
16	Arginine	$C_6H_{14}N_4O_2$	0.9357	175.106	174.1117	down
17	L-Histidine	$C_6H_9N_3O_2$	1.214	155.0719	155.0695	down
18	L-Serine	$C_3H_7NO_3$	0.6959	105.042	105.0426	down
19	2-Amino-5-oxohexanoic acid	$C_6H_{11}NO_3$	0.6456	163.1064	145.0739	up
20	D-Glutamic acid	$C_5H_9NO_4$	0.9171	189.0857	147.0532	up
21	Pyrrole-2-carboxylic acid	$C_5H_5NO_2$	8.9184	196.0017	111.0320	up
22	L-Proline	$C_5H_9NO_2$	0.9294	116.0697	115.0633	up
23	Phenylpyruvic acid	$C_9H_8O_3$	3.3828	163.0407	164.0473	up
24	L-Aspartic acid	$C_4H_7NO_4$	1.0529	134.0436	133.0375	up
25	D-Ornithine	$C_5H_{12}N_2O_2$	1.2571	115.0859	132.0899	up
26	L-Alanine	$C_3H_7NO_2$	0.948	90.0361	89.0477	up
27	L-Threonine	$C_4H_9NO_3$	0.9294	120.0646	119.0582	up
28	N-Acetyl-L-glutamic acid	$C_7H_{11}NO_5$	1.2263	190.0695	189.0637	up
29	5-Aminopentanoic acid	$C_5H_{11}NO_2$	1.8805	116.0714	117.079	up
30	cis-4-Hydroxy-D-proline	$C_5H_9NO_3$	0.955	171.0779	131.0582	up
31	Oxalacetic acid	$C_4H_4O_5$	6.0386	191.0364	132.0059	down
32	D-Pyroglutamic acid	$C_5H_7NO_3$	1.3252	147.0751	129.0426	up
33	D-Alanine	$C_3H_7NO_2$	1.3397	90.0547	89.0477	up
34	D-phenylalanine	$C_9H_{11}NO_2$	4.1789	166.0851	165.079	up
35	L-Isoleucine	$C_6H_{13}NO_2$	1.2759	132.1007	131.0946	up
36	L-Leucine	$C_6H_{13}NO_2$	1.5107	132.1007	131.0946	up
37	Ketonuleucine	$C_6H_{10}O_3$	2.8957	129.0667	130.063	up
38	3-Isopropylmalic acid	$C_7H_{12}O_5$	2.8086	216.0881	176.0685	up

39	(S)-2-Aceto-2-hydroxybutanoic acid	$C_6H_{10}O_4$	1.6933	127.0399	146.0579	up
40	3-Hydroxy-3-methyl-2-oxobutanoic acid	$C_5H_8O_4$	1.2103	131.0459	132.0423	up
41	(R)-2,3-Dihydroxy-isovalerate	$C_5H_{10}O_4$	1.7244	267.1111	134.0579	up
42	L-Argininosuccinic acid	$C_{10}H_{18}N_4O_6$	1.6364	290.1319	290.1226	up
43	Citrulline	$C_6H_{13}N_3O_3$	0.9357	176.1016	175.0957	up
44	N-Acetyl-L-glutamic acid	$C_7H_{11}NO_5$	1.2263	190.0695	189.0637	up
45	N-acetylornithine	C7H14N2O3	1.0659	173.093	174.1004	down
46	Ureidosuccinic acid	$C_5H_8N_2O_5$	0.6896	223.0567	176.0433	down
47	Spasmic acid	$C_{11}H_{16}N_2O_8$	0.6838	303.0888	304.0907	up
48	N-Acetyl-L-aspartic acid	$C_6H_9NO_5$	1.215	174.0407	175.0481	up
49	Asparagine	$C_4H_8N_2O_3$	0.8863	133.0595	132.0535	down
50	Tryptamine	$C_{10}H_{12}N_2$	2.8016	161.1055	160.1026	down
51	2-Aminophenol	C_6H_7NO	1.449	110.0595	109.0528	up
52	Xanthurenic acid	$C_{10}H_7NO4$	3.1031	206.0388	205.0375	down
53	Melatonin	$C_{13}H_{16}N_2O_2$	5.3338	197.1156	232.1212	up
54	3-Hydroxyanthranilic acid	$C_7H_7NO_3$	1.475	153.0334	153.0426	down
55	L-Tryptophan	$C_{11}H_{12}N_2O_2$	2.5325	205.0958	204.0899	up
56	Indoleacetic acid	$C_{10}H_9NO_2$	4.5734	174.0566	175.0633	up
57	5-Hydroxyindoleacetic acid	$C_{10}H_9NO_3$	3.5692	190.0519	191.0582	up
58	3-Hydroxy-3-methyloxindole	$C_9H_9NO_2$	2.3459	222.0979	163.0633	up
59	2-Aminobenzoic acid	$C_7H_7NO_2$	2.5369	120.0435	137.0477	up
60	5-Hydroxyindoleacetaldehyde	$C_{10}H_9NO_2$	4.0218	174.0565	175.0633	up
61	Picolinic acid	$C_6H_5NO_2$	9.7796	124.0384	123.0320	up
62	Cinnabarinic acid	$C_{14}H_8N_2O_6$	3.2207	340.0552	300.0382	up
63	β-Alanine	$C_3H_7NO_2$	1.214	158.0434	89.0477	down
64	Pantetheine	$C_{11}H_{22}N_2O_4S$	2.1834	279.1304	278.13	up
65	Panthenol	$C_9H_{19}NO_4$	3.3024	188.1251	205.1314	up
66	Uracil	$C_4H_4N_2O_2$	0.6839	113.0348	112.0273	up
67	Pantothenic acid	$C_9H_{17}NO_5$	2.3213	220.1159	219.1107	up
68	Pantoic acid	$C_6H_{12}O_4$	1.4833	147.0668	148.0736	up
69	(R)-2,3-Dihydroxy-isovalerate	$C_5H_{10}O_4$	1.7244	267.1111	134.0579	up
70	2-dehydropantoate	$C_6H_{10}O_4$	2.6088	198.054	146.0579	down
71	alpha-D-Galactose 1-phosphate	$C_6H_{13}O_9P$	0.6829	225.0181	260.0297	down
72	Sucrose	$C_{12}H_{22}O_{11}$	0.9232	707.2162	342.1162	down
73	Mannose	$C_6H_{12}O_6$	0.8192	180.0669	180.0634	up
74	Sorbitol	$C_6H_{14}O_6$	1.7606	182.0659	182.079	up
75	Tagatose	$C_6H_{12}O_6$	1.1284	179.0564	180.0634	up
76	Stachyose	$C_{24}H_{42}O_{21}$	1.1026	689.2032	666.2219	down
77	D-Fructose-6-phosphate	$C_6H_{13}O_9P$	0.7256	319.0451	260.0297	down
78	Uridine-5'-diphosphate-glucose	$C_{15}H_{24}N_2O_{17}P_2$	1.218	565.0506	566.055	down
79	L-Tyrosine	C9H11NO3	1.2263	182.0612	181.0739	up
80	4-Hydroxyphenylpyruvic acid	$C_9H_8O_4$	4.0782	180.0429	180.0423	up
81	L-Homophenylalanine	$C_{10}H_{13}NO_2$	2.9642	197.127	179.0946	up
82	3-Hydroxybenzoic acid	$C_7H_6O_3$	3.8451	137.0245	138.0317	up

Table S6. Identification of differential metabolites (C vs. A)

No.	Metabolite	Formula	Retention time (min)	Precursor (g/mol)	Mass (g/mol)	Trend
1	Glutamine	$C_5H_{10}N_2O_3$	0.9171	147.0751	146.0691	down
2	Citric acid	$C_6H_8O_7$	0.8572	192.0227	192.0270	up
3	Succinic acid semialdehyde	$C_4H_6O_3$	1.1337	140.9935	102.0317	down
4	Ureidosuccinic acid	$C_5H_8N_2O_5$	0.6896	223.0567	176.0433	down
5	gamma-Aminobutyric acid	$C_4H_9NO_2$	2.8503	86.0598	103.0633	up
6	Succinic acid	$C_4H_6O_4$	0.9673	117.0189	118.0266	down
7	L-Alanine	$C_3H_7NO_2$	1.598	88.04	89.0477	up
8	N-Acetyl-L-aspartic acid	$C_6H_9NO_5$	1.215	174.0407	175.0481	down
9	Asparagine	$C_4H_8N_2O_3$	3.3707	263.1057	132.0535	up
10	α-Ketoglutaramate	$C_5H_7NO_4$	0.8192	126.0193	145.0375	up
11	Nicotinic acid	$C_6H_5NO_2$	0.6584	123.0398	123.0320	down
12	Nicotinamide mononucleotide	$C_{11}H_{15}N_2O_8P$	3.2027	335.0647	334.0566	up
13	Maleic acid	$C_4H_4O_4$	1.2103	115.0035	116.0110	down
14	Cytosine	$C_4H_5N_3O$	1.0036	112.0497	111.0433	down
15	β-Alanine	$C_3H_7NO_2$	1.214	158.0434	89.0477	up
16	Orotic acid	$C_5H_4N_2O_4$	0.6885	157.0270	156.0171	down
17	4-Hydroxytoluene sulfonamide	$C_{12}H_{18}N_2O_4S$	5.0205	309.0838	286.0987	down
18	Cytidine-5'-triphosphate	$C_9H_{16}N_3O_{14}P_3$	0.8062	501.0209	482.9845	up
19	Deoxyuridine-5'-diphosphate	$C_9H_{14}N2O_{11}P_2$	0.6635	389.0101	388.0073	up
20	Uracil	$C_4H_4N_2O_2$	1.2273	111.0084	112.0273	down
21	Thymidine	$C_{10}H_{14}N_2O_5$	1.4502	241.0843	242.0903	down
22	Thymine	$C_5H_6N_2O_2$	1.215	185.0574	126.0429	down
23	L-Dihydroorotic acid	$C_5H_6N_2O_4$	0.8192	198.0504	158.0328	down
24	Deoxycytidine diphosphate	$C_9H_{15}N_3O_{10}P_2$	0.6592	368.0073	387.0233	up
25	Uridine-5'-diphosphate	$C_9H_{14}N_2O_{12}P_2$	1.2211	402.9983	404.0022	down
26	Carbamic acid	CH_3NO_2	2.0292	120.0379	61.0164	up
27	Ureidoperacrylic acid	$C_4H_6N_2O_4$	0.7823	162.049	146.0328	down
28	Ureidoacrylic acid	$C_4H_6N_2O_3$	7.3382	165.0015	130.0378	down
29	Maltose	$C_{12}H_{22}O_{11}$	0.9427	683.2326	342.1162	down
30	Sucrose	$C_{12}H_{22}O_{11}$	1.3509	401.1335	342.1162	down
31	Trehalose-6-phosphate	$C_{12}H_{23}O_{14}P$	0.733	421.0766	422.0825	down
32	D-Fructose-6-phosphate	$C_6H_{13}O_9P$	0.7256	319.0451	260.0297	down
33	Uridine-5'-diphosphate-glucose	$C_{15}H_{24}N_2O_{17}P_2$	1.218	565.0506	566.055	down
34	2-Aminophenol	C_6H_7NO	1.449	110.0595	109.0528	up
35	5,5'-Dehydrodivanillate	$C_{16}H_{14}O_8$	5.1507	299.0636	334.0689	up
36	1,2,3-Trihydroxybenzene	$C_6H_6O_3$	6.7595	109.0219	126.0317	down
37	4-Nitrocatechol	$C_6H_5NO_4$	2.5989	156.0372	155.0219	down
38	2-Pyrocatechuic acid	$C_7H_6O_4$	2.5842	153.0192	154.0266	up

39	4-Hydroxybenzoic acid	$C_7H_6O_3$	3.4148	137.0245	138.0317	up
40	Mandelic acid	$C_8H_8O_3$	3.2207	151.0401	152.0473	up
41	Phenylglyoxylic acid	$C_8H_6O_3$	2.6215	149.0279	150.0317	down
42	Gallic acid	$C_7H_6O_5$	5.7647	229.0452	170.0215	up
43	2-Aminobenzoic acid	$C_7H_7NO_2$	3.2644	273.0894	137.0477	up
44	Glucosamine	$C_6H_{13}NO_6$	1.1645	178.0698	195.0743	down
45	3-Phosphoglyceric acid	$C_3H_7O_7P$	0.6763	186.9935	185.9929	up
46	Gluconic Acid	$C_6H_{12}O_7$	1.0351	195.0532	196.0583	down
47	6-Phosphogluconic acid	$C_6H_{13}O_{10}P$	0.7207	297.0008	276.0246	down
48	Ribulose 5-phosphate	$C_5H_{11}O_8P$	0.733	229.0135	230.0192	down
49	D-Xylulose 5-phosphate	$C_5H_{11}O_8P$	0.8315	289.0355	230.0192	down
50	Ribose-1-phosphate	$C_5H_{11}O_8P$	1.1777	275.0186	230.0192	down
51	D-Ribofuranose 5-phosphate	$C_5H_{11}O_8P$	1.1668	211.0024	230.0192	down
52	L-glyceraldehyde	$C_3H_6O_3$	0.8271	159.026	90.0317	down
53	D-Xylulose 5-phosphate	$C_5H_{11}O_8P$	0.8315	289.0355	230.0192	down
54	Digalacturonate	$C_{12}H_{18}O_{13}$	4.5734	369.0665	370.0747	up
55	Dihydroxyacetone phosphate	$C_3H_7O_6P$	1.1284	229.0135	169.998	down
56	β-Alanine	$C_3H_7NO_2$	1.214	158.0434	89.0477	up
57	Panthenol	$C_9H_{19}NO_4$	3.3024	188.1251	205.1314	up
58	3-Aminopropionaldehyde	C_3H_7NO	0.9171	96.0442	73.0528	up
59	Uracil	$C_4H_4N_2O_2$	1.2273	111.0084	112.0273	down
60	alpha-Ketoisovaleric acid	$C_5H_8O_3$	2.821	115.0399	116.0473	up
61	Pantothenic acid	$C_9H_{17}NO_5$	2.3212	437.2162	219.1107	up
62	2-dehydropantoate	$C_6H_{10}O_4$	2.6088	198.054	146.0579	down
63	Phosphoenolpyruvate	$C_3H_5O_6P$	0.5602	148.9672	167.9824	up
64	Malic acid	$C_4H_6O_5$	1.1366	133.014	134.0215	down
65	Isocitric acid	$C_6H_8O_7$	0.8192	191.0203	192.0270	up
66	alpha-D-Galactose 1-phosphate	$C_6H_{13}O_9P$	0.6829	225.0181	260.0297	down
67	Stachyose	$C_{24}H_{42}O_{21}$	0.7699	701.1987	666.2219	down
68	D-Gal alpha 1-6D-Gal alpha 1-6D-Glucose	$C_{18}H_{32}O_{16}$	1.1654	503.1652	504.1690	down

Table S7. Mobile phase gradient program for the T3 column

**Time (min)**	**A (%)**	**B (%)**
0.0	95	5
2.0	80	20
5.0	40	60
6.0	1	99
7.5	1	99
7.6	95	5
10.0	95	5

Table S8. Values of ANI and dDDH between F204 and its closest species

Type strain	ANI (%)	dDDH (%)
Streptomyces fodineus TW1S1^{T}	90.43	41.40
Streptomyces camelliae HUAS26^{T}	88.14	35.50

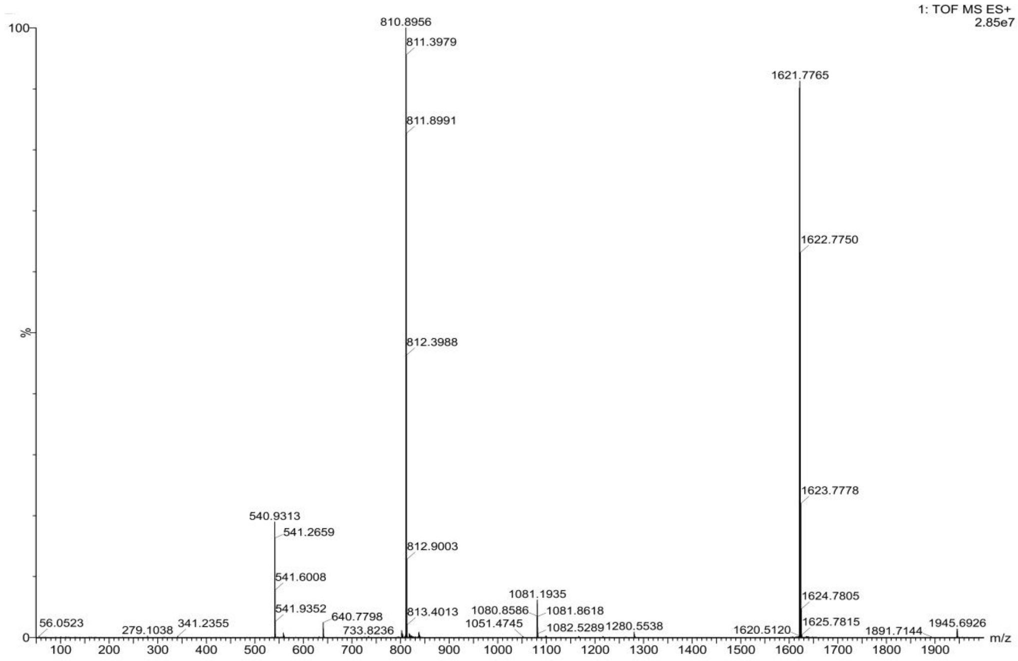

Figure S1 Mass spectrum of compound 1

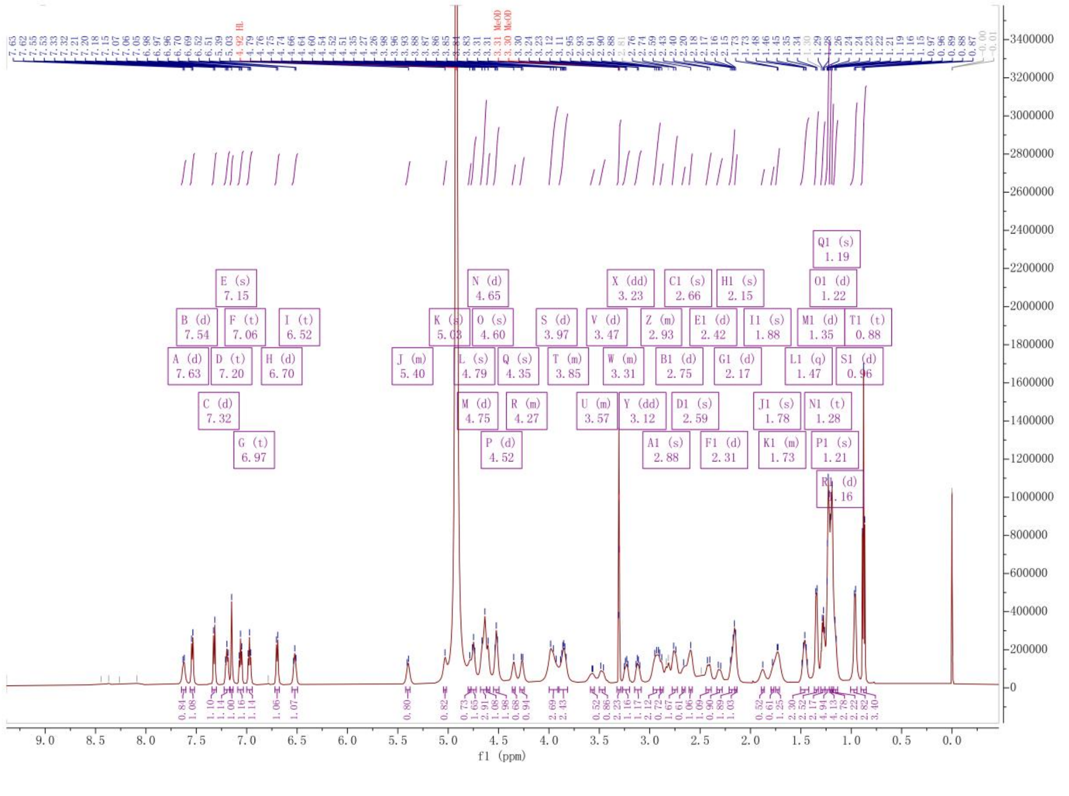

Figure S2. ^{1}H NMR spectrum of compound 1

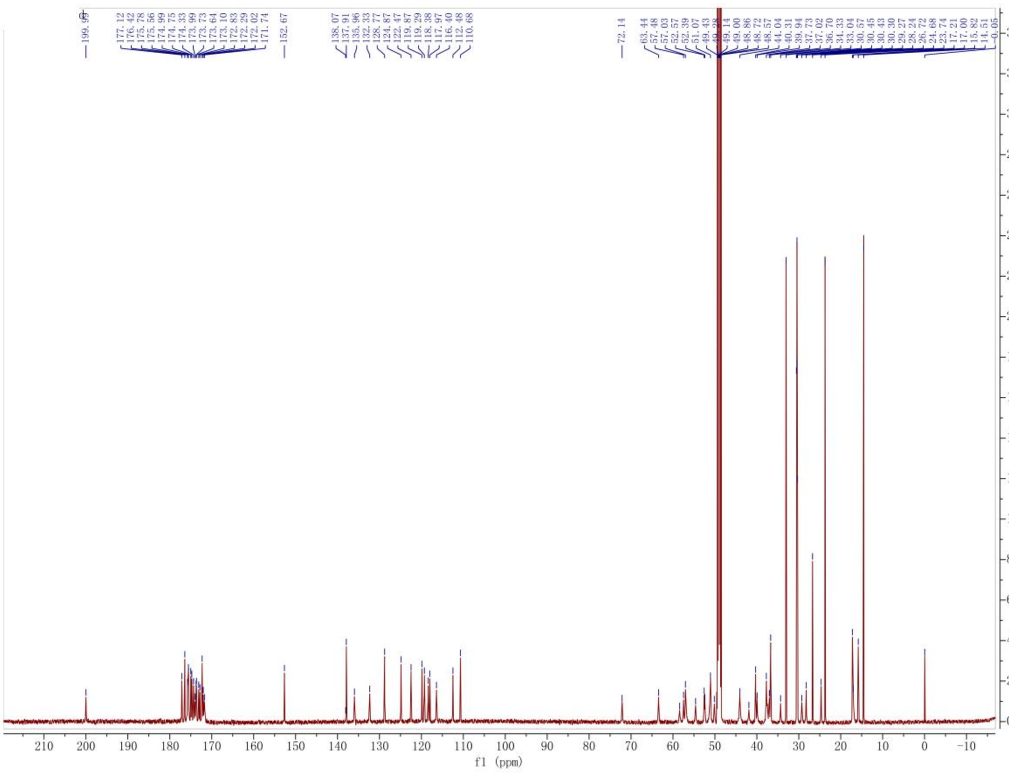

Figure S3. ^{13}C NMR spectrum of compound 1

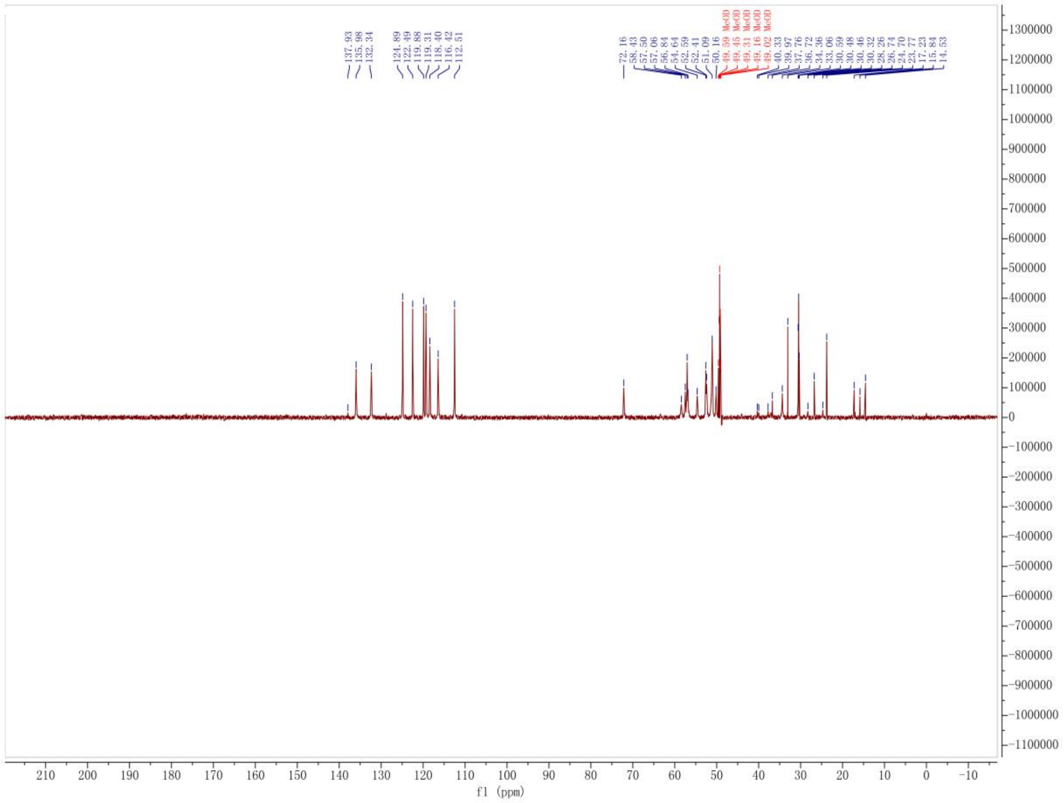

Figure S4. DEPT-90° spectrum of compound 1

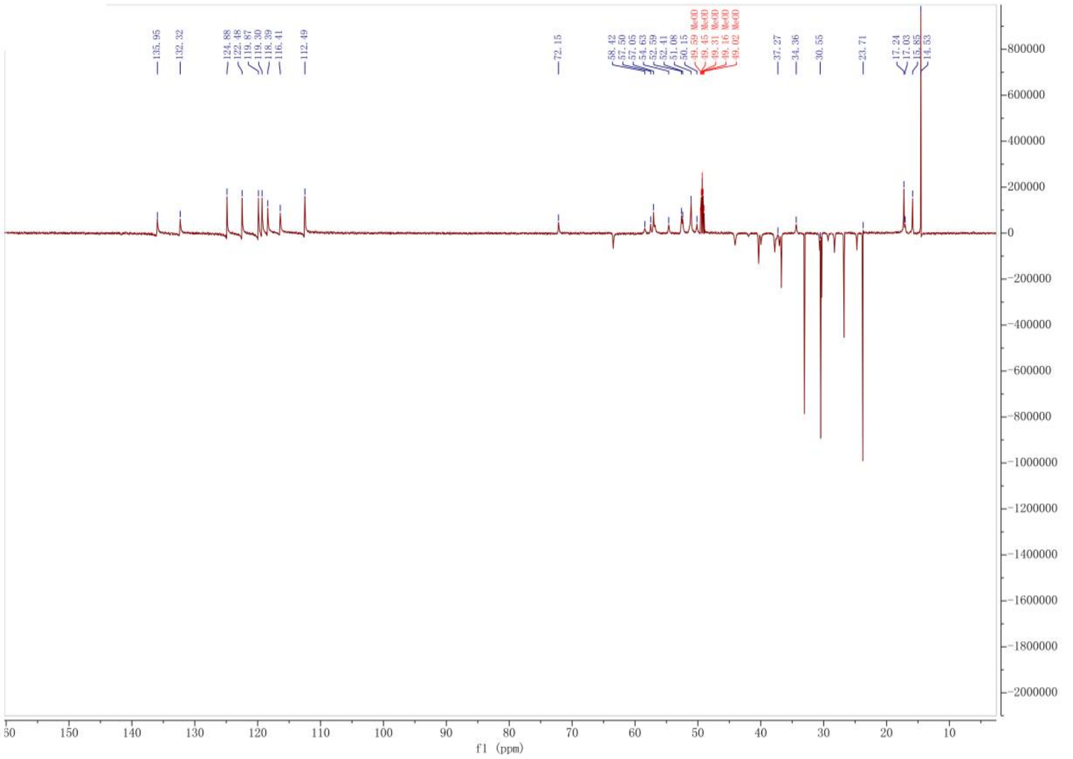

Figure S5. DEPT-135° spectrum of compound 1

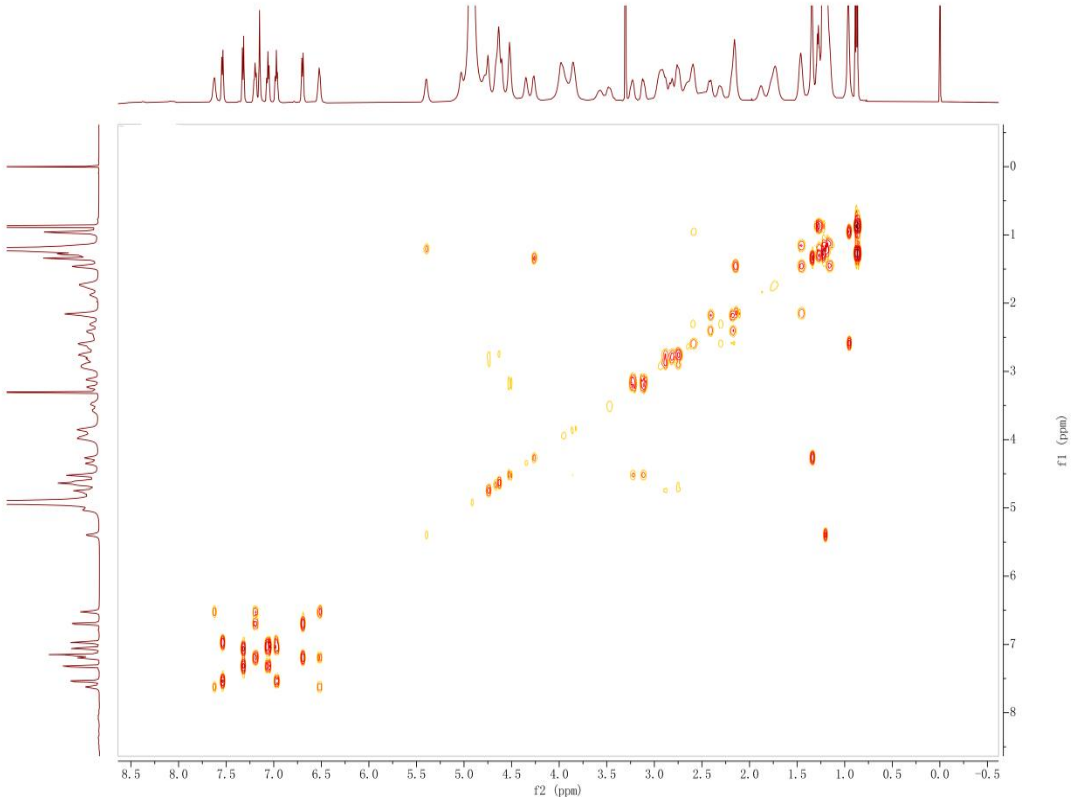

Figure S6. 1H-1H COSY spectrum of compound 1

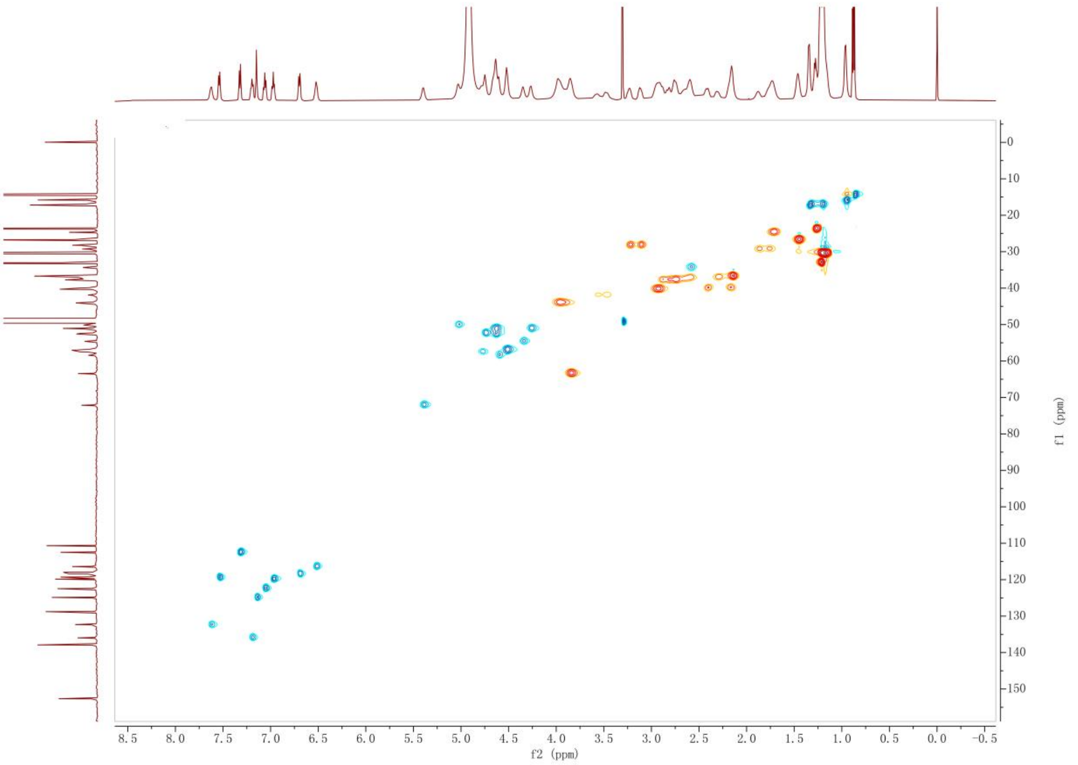

Figure S7. HSQC spectrum of compound 1

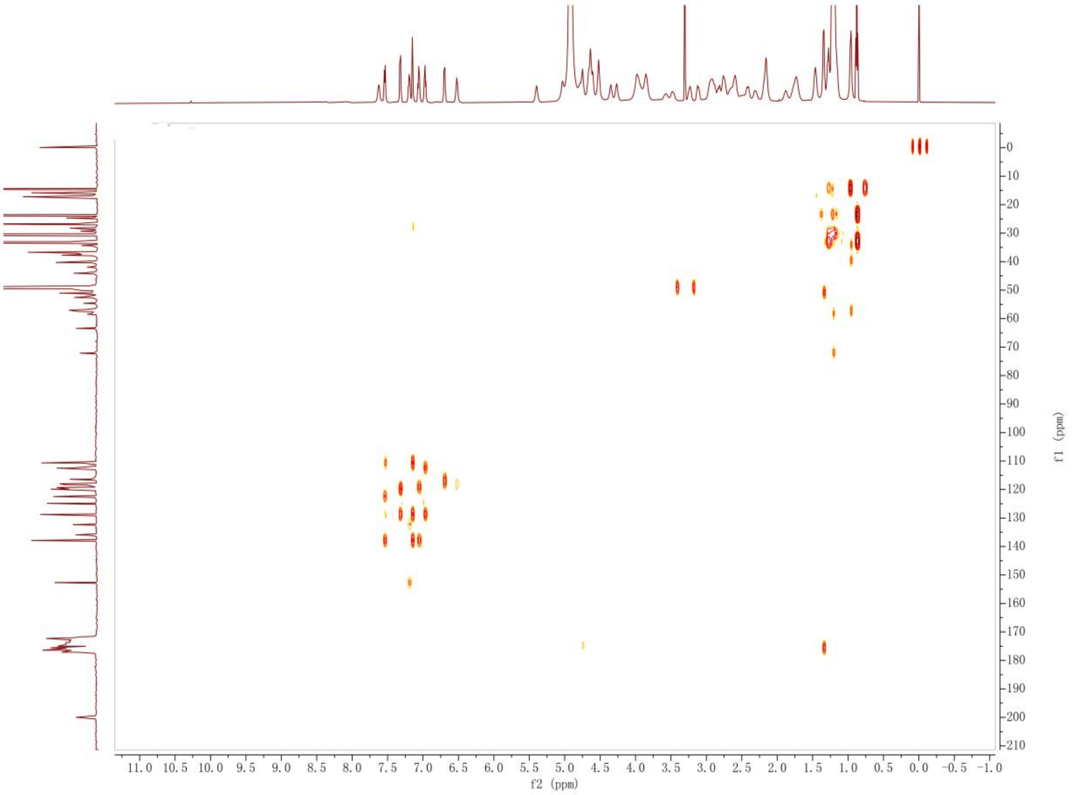

Figure S8. HMBC spectrum of compound 1

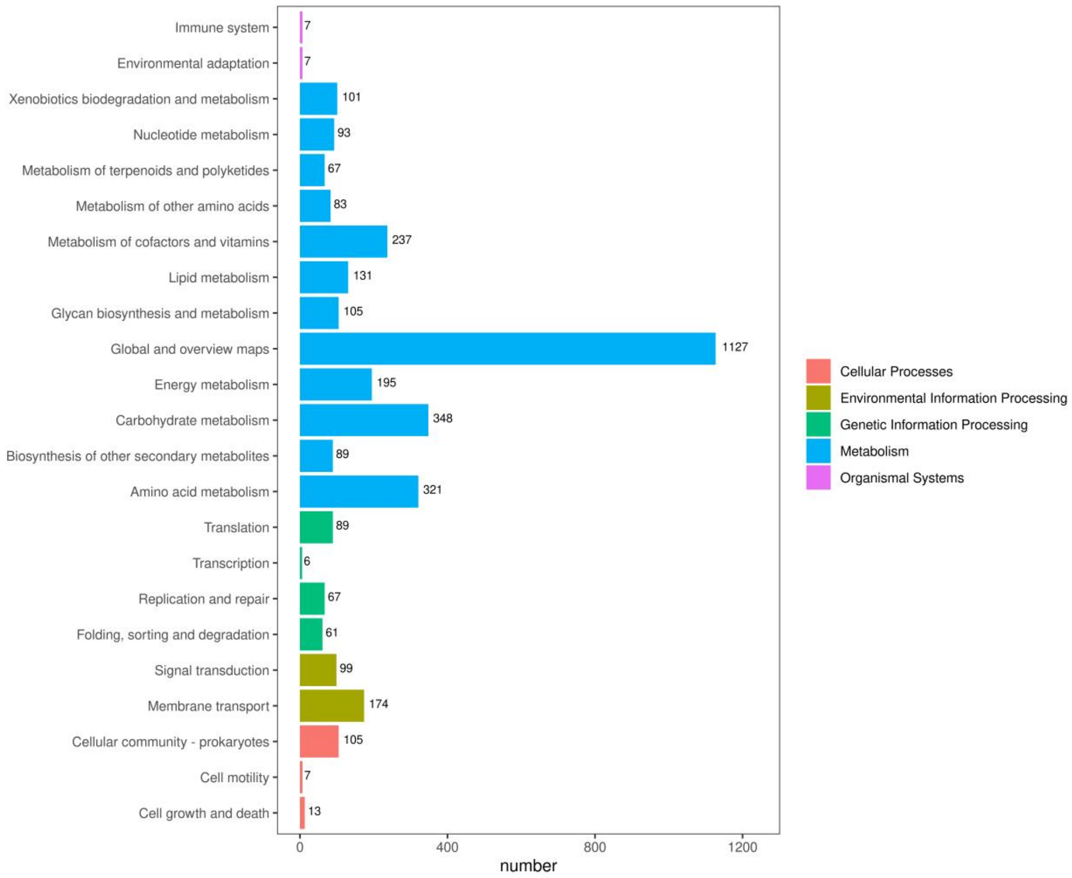

Figure S9. Distribution of Streptomyces sp.F204 KEGG functional annotations.

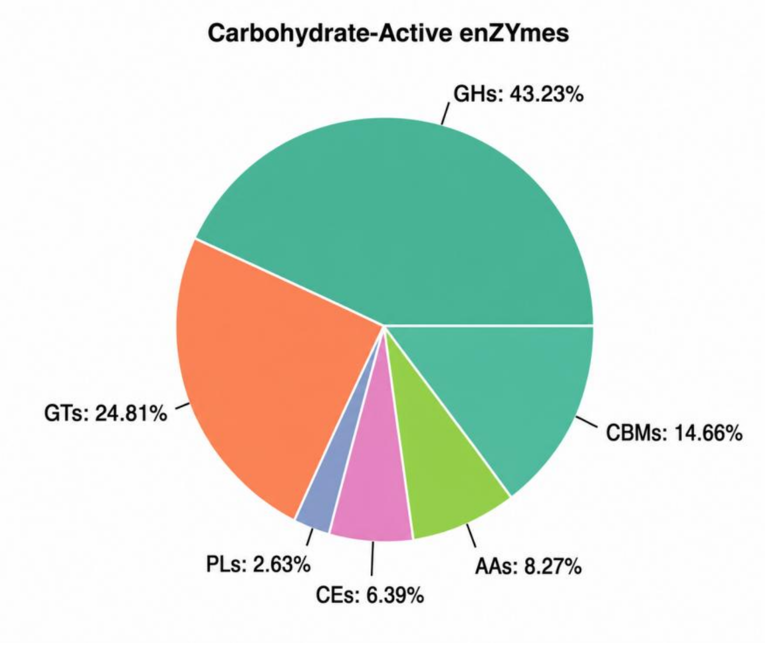

Figure S10. A pie chart illustrates the CAZymes encoded

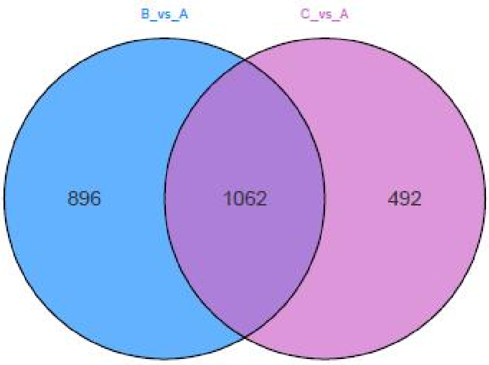

Figure S11. Venn diagram of differential metabolites among groups
